# Supplementary material for: Discovery of New Coumarin-Based Lead with Potential Anticancer, CDK4 Inhibition and Selective Radiotheranostic Effect: Synthesis, 2D & 3D QSAR, Molecular Dynamics, In Vitro Cytotoxicity, Radioiodination, and Biodistribution Studies
Source: Molecules. 2021 Apr 14;26(8):2273. doi: 10.3390/molecules26082273 (PMC8070829; doi:10.3390/molecules26082273)
Supplement: Supplementary file 1 [file molecules-26-02273-s001.pdf]

Supporting information

Discovery of new coumarin-based lead with potential anticancer, CDK4 inhibition and selective radiotheranostic effect: Synthesis, 2D & 3D *QSAR*, Molecular dynamics, *in vitro* cytotoxicity, radioiodination and biodistribution studies

Mona O. Sarhan[1] Somaia S. Abd El-Karim[2] Manal M. Anwar[2] Raghda H. Gouda[3] Wafaa A. Zaghary[3*] Mohammed A. Khedr[3,*]

[1] Labelled compounds department, Hot lab Centre, Egyptian Atomic Energy Authority; monasarhan@windowslive.com
[2] Department of Therapeutic Chemistry, National Research Centre, Dokki, Cairo 12622, Egypt; somaia_elkarim@hotmail.com; manal.hasan52@live.com
[3] Department of Pharmaceutical Chemistry, Faculty of Pharmacy, Helwan University, P.O. Box 11795, Cairo, Egypt; raghdahassan@yahoo.com; wafaa.zaghary@pharm.helwan.edu.eg; mohammed_abdou@pharm.helwan.edu.eg
* Correspondence: Prof. Dr./ Wafaa Zaghary: Wafaa.zaghary@pharm.helwan.edu.eg; Associate prof./ Mohammed Abdou: mohammed_abdou@pharm.helwan.edu.eg

Corresponding authors:

Prof. Dr./ Wafaa Zaghary: Wafaa.zaghary@pharm.helwan.edu.eg

Associate prof./ Mohammed Abdou: mohammed_abdou@pharm.helwan.edu.eg

Chemical synthesis and spectroscopic data

2-(1-(6-Bromo-2-oxo-2H-chromen-3-yl)ethylidene)-N-methyl/phenylhydrazine-1-carbothioamide **2a,b**

To a hot solution of the 3-acetyl-6-bromo-2H-chromen-2-one (1.8 mmol) in absolute ethanol (10 ml), 4-methyl/phenyl-thiosemicarbazide (1.8 mmol) and 5 drops of concentrated HCl were added. The mixture was refluxed for 14-17 h. The reaction mixture was allowed to cool, the formed precipitate was filtered, dried and recrystallized from absolute ethanol to afford the title compounds **2a,b**.

2-(1-(6-Bromo-2-oxo-2H-chromen-3-yl) ethylidene)-N-methylhydrazine-1-carbothioamide (**2a**)

Yield 66%; white powder; m.p. 220°C; crystalized from ethanol; IR (KBr, υ cm^{-1}): 3449 (2NH), 1736 (C=O), 1674 (C=N); ^{1}H NMR (DMSO-d6): δ 2.58 (s, 3H, CH_3), 3.75 (s, 3H, CH_3), 6.91 (d, 1H, Ar-H, J=7.6 Hz), 7.30-7.45 (m, 2H, Ar-H), 7.85 (s, 1H, NH, D2O exchangeable), 8.18 (s, 1H, Ar-H), 8.59 (s, 1H, Ar-H), 9.57 (s, 1H, NH, D2O exchangeable); ^{13}C NMR (DMSO-d6): δ 30.41 (CH_3), 55.68 ($NHCH_3$), 116.84, 118.93, 120.46, 126.67, 132.93,135.27, 146.11, 154.03, 154.36 (Ar-C), 158.43 (C=O, coumarin), 195.55 (C=S); MS (EI) m/z (%): 353, 355 (M+, 85, 100); Anal. Calcd. For $C_{13}H_{12}BrN_3O_2S$ (354.22): C, 44.08; H, 3.41; Br, 22.56; N, 11.86; O, 9.03; S, 9.05; Found: C, 44.31; H, 3.65; N, 11.70.

Supporting information

2-(1-(6-Bromo-2-oxo-2H-chromen-3-yl)ethylidene)-N-phenylhydrazine-1-carbothioamide (**2b**)

yield 53%; orange powder; m.p.194°C; crystalized from ethanol; IR (KBr, υ cm^{-1}): 3449 (2NH), 1728 (C=O), 1628 (C=N); ^{1}H NMR (DMSO-d6) δ: 2.34 (s, 3H, CH_3), 7.18-7.78 (m, 7H, Ar-H), 7.97 (s, 1H, Ar-H), 8.42 (s, 1H, Ar-H), 10.10 (s, 1H, NH, D2O exchangeable), 10.89 (s, 1H, NH, D2O exchangeable); ^{13}C NMR (DMSO-d6): δ 16.02 (CH_3), 116.20, 118.21, 120.72, 125.12, 125.36, 125.88, 126.52, 128.20, 129.86, 130.99, 134.63, 138.84, 140.82, 145.85, 152.32 (Ar-C), 158.43 (C=O, coumarin), 176.99 (C=S); MS (EI) m/z (%): 417 (M++1, 3), 324 (M+, 100); Anal. Calcd. For $C_{18}H_{14}BrN_3O_2S$ (416.29): C, 51.93; H, 3.39; Br, 19.19; N, 10.09; O, 7.69; S, 7.70; Found: C, 52.09; H, 3.62; N, 10.25.

2-((1-(6-Bromo-2-oxo-2H-chromen-3-yl)ethylidene)hydrazino)-3-methyl/phenyl-thiazolidin-4-one **3a,b**

To a hot solution of the 2a,b (1.4 mmol) in absolute ethanol (20 mL), ethyl bromoacetate (0.2g, 1.5 mmol) and anhydrous sodium acetate (0.2g, 2.8 mmol) were added. The reaction mixture was refluxed for 7-12 h. The formed precipitate was filtered, washed with water, dried and recrystallized from the appropriate solvent to afford the corresponding title compound **3a,b**.

2-((1-(6-Bromo-2-oxo-2H-chromen-3-yl)ethylidene)hydrazono)-3-methylthiazolidin-4-one (**3a**)

Yield 60%; canary yellow powder; m.p. 250-252°C; crystalized from ethanol; IR (KBr, υ max / cm^{-1}): 1744, 1700 (2C=O), 1636 (2C=N); ^{1}H NMR (DMSO-d6): δ 2.35 (s, 3H, CH_3), 3.20 (s, 3H, NCH_3), 3.97 (s, 2H, CH_2-thiazolidine), 7.42 (d, 1H, Ar-H, J =8.00 Hz,), 7.79 (d, 1H, Ar-H, J = 8.00 Hz), 8.15 (s, 1H, Ar-H), 8.17 (s, 1H, Ar-H); ^{13}C NMR (DMSO-d6): δ 17.3 (CH_3), 29.9 (CH_2), 32.6 (NCH_3), 116.77, 118.76, 121.06 , 127.90, 131.73, 135.33, 140.81, 153.03, 159.00, 160.92 (C=O coumarin), 165.23 (C=N), 172.76 (C=O thiazolidine); MS (EI) m/z (%): 392.78, 394.89 (M+, 74, 78), 320, 322 ($C_{13}H_{11}BrN_3O_2$, 100, 88); Anal. Calcd. for $C_{15}H_{12}BrN_3O_3S$ (394.24): C, 45.70; H, 3.07, N, 10.66; S, 8.13; Found: C, 45.92; H, 3.23; N, 10.89.

2-((1-(6-Bromo-2-oxo-2H-chromen-3-yl)ethylidene)hydrazono)-3-phenylthiazolidin-4-one (**3b**)

Yield 60%; yellow powder; m.p. 275-277°C; crystalized from glacial acetic acid; IR (KBr, υ cm^{-1}): 1726, 1670 (2C=O), 1620 (2C=N); ^{1}H NMR (DMSO-d6): δ 2.09 (s, 3H, CH_3), 4.12 (s, 2H, CH_2 thiazolidine), 7.40-7.48 (m, 4H, Ar-H), 7.51-7.55 (m, 2H, Ar-H), 7.80 (dd, J = 2, 8 Hz, 1H, Ar-H), 8.15 (s, 1H, Ar-H), 8.16 (s, 1H, Ar-H); ^{13}C NMR (DMSO-d6,75 MHz) δ ppm: 17.27 (CH_3), 32.95 (CH_2), 116.83, 118.79, 120.72, 128.48, 129.07, 129.43, 131.77, 135.39, 140.89, 152.95, 154.01, 161.00 (C=O coumarin), 164.00 (C=N), 172.38 (C=O thiazolidine); MS (EI) m/z (%): 455.11, 457.11 (M+, 100, 98); Anal. Calcd. for $C_{20}H_{14}BrN_3O_3S$ (456.31): C, 52.64; H, 3.09; N, 9.21; S, 7.03; Found: C, 52.80; H, 3.23; N, 9.44; S, 7.33.

6-Bromo-3-(1-((3-methyl/phenyl-4-phenylthiazol-2(3H)ylidene)hydrazineylidene) ethyl)-2H-chromen-2-one **4a,b**

To a hot solution of the 6-bromocoumarin compound **2a,b** (1.4 mmol) in absolute ethanol (20 mL), phenacylbromide (0.3g, 1.4mmol) and anhydrous sodium acetate (0.2g, 2.8mmol) were added. The reaction

mixture was refluxed for 9h. The formed precipitate was filtered then washed with water, filtered, dried and recrystallized from the proper solvent to afford the corresponding the title compounds 4a,b.

6-Bromo-3-(1-((3-methyl-4-phenylthiazol-2(3H)-ylidene)hydrazono)ethyl)-2H-chromen-2-one (**4a**)

Yield 57 %; orange powder powder; m.p. 200 °C; crystalized from ethanol; IR (KBr, υ cm^{-1}): 1744 (C=O), 1636 (2C=N); ^{1}H NMR (DMSO-d6): δ 2.34 (s, 3H, CH_3), 3.35 (s, 3H, CH_3, masked by H2O), 6.46 (s, 1H, CH-thiazole proton), 7.38 (d, 1H, Ar-H, J=11.2 Hz), 7.49-7.55 (s, 5H, Ar-H), 7.76 (dd, 1H, Ar-H, J=3.2, 8.4 Hz,), 8.12 (s, 1H, Ar-H), 8.14 (d, 1H, Ar-H, J=4.0 Hz,); ^{13} C NMR (DMSO-d6): δ 16.91 (CH_3), 34.07 (NCH_3), 100.76, 116.67, 118.60, 121.48, 128.58, 129.17, 129.27, 129.67, 130.92, 131.44, 134.60, 139.12, 141.05, 152.76, 152.84, 159.35, 170.55; MS (EI) m/z (%): 453.17, 455.11 (M•+, 100, 97); Anal. Calcd. for $C_{21}H_{16}BrN_3O_2S$ (454.34) : C, 55.51; H, 3.55; Br, 17.59; N, 9.25; O, 7.04; S, 7.06; Found: C, 55.68; H, 3.49; N, 9.43.

6-Bromo-3-(1-((3,4-diphenylthiazol-2(3H)-ylidene)hydrazono)ethyl)-2H-chromen-2-one (**4b**)

Yield 50%; yellow powder; m.p. 234°C; crystalized from glacial acetic acid; IR (KBr, υ cm^{-1}): 1736 (C=O), 1597 (2C=N); ^{1}H NMR (DMSO-d6): δ 2.12 (s, 3H, CH_3), 6.69 (s, 1H, CH-thiazole proton), 7.17-7.19 (m, 2H, Ar-H), 7.22-7.32 (m, 6H, Ar-H), 7.36-7.40 (m, 3H, Ar-H), 7.76 (dd, 1H, Ar-H, J=3.2, 8.8 Hz,), 8.12 (s, 2H, Ar-H); ^{13}C NMR (DMSO-d6) δ: 16.98 (CH_3), 102.60, 116.69, 118.64, 121.38, 128.26, 128.65, 128.74, 128.89, 129.22, 131.17, 131.50, 134.78, 138.10, 139.56, 140.11, 152.81, 154.46, 159.18, 170.50; MS (EI) m/z (%): 515.13, 517.00 (M•+, 69, 100); Anal. Calcd. for $C_{26}H_{18}BrN_3O_2S$ (516.41): C, 60.47; H, 3.51; N, 8.14; S, 6.21; Found: C, 60.31; H, 3.69; N, 8.35; S, 6.02.

Bromo-3-(1-((4-(4-bromophenyl)-3-methyl/phenylthiazol-2(3H)-ylidene) hydrazono)ethyl)-2H-chromen-2-one **5a,b**

To a hot solution of compound **2a,b** (1.2 mmol) in absolute ethanol (20 mL), p-bromophenacyl bromide (0.3g, 1.2mmol) and anhydrous sodium acetate (0.2g, 2.4mmol) were added. The reaction mixture was refluxed for 19h. After reaction completion, the formed precipitate was filtered while hot then washed with water, filtered, dried and crystalized from the proper solvent to accomplish the corresponding derivatives 5a,b.

6-Bromo-3-(1-((4-(4-bromophenyl)-3-methylthiazol-2(3H)-ylidene)hydrazono)ethyl)-2H-chromen-2-one (**5a**)

Yield 43%; orange powder; m.p.251°C; crystalized from ethanol; IR (KBr, υ cm^{-1}): 1728 (C=O), 1636 (2C=N); ^{1}H NMR (DMSO-d6): δ 2.33 (s, 3H, CH_3), 3.35 (s, 3H, CH_3), 6.52 (s, 1H, CH-thiazole proton), 7.40 (d, 1H, Ar-H, J=8.0 Hz,), 7.48 (d, 2H, Ar-H, J=12.0 Hz), 7.70 (d, 2H, Ar-H, J=8.0 Hz), 7.76.7.78 (m, 1H, Ar-H), 8.13 (s, 1H, Ar-H), 8.15 (s, 1H, Ar-H); ^{13}C NMR (DMSO-d6): δ 19.24 (CH_3), 34.99 (NCH_3), 102.00, 117.23, 118.00, 122.00, 131.58, 132.94, 135.26, 139.64, 151.00, 153.50 (Ar-C), 160.0 (C=O), 170.5 (C=N); MS (EI) m/z (%): 531.11, 533.14, 535.14 (M+, 45, 100, 50); Anal. Calcd. for $C_{21}H_{15}Br_2N_3O_2S$ (533.24): C, 47.30; H, 2.84; N, 7.88; S, 6.01; Found: C, 47.52; H, 3.11; N, 8.12; S, 6.37.

Supporting information

6-Bromo-3-(1-((4-(4-bromophenyl)-3-phenylthiazol-2(3H)-ylidene)hydrazono)ethyl)-2H-chromen-2-one (**5b**)

Yield 43%; orange powder; m.p. 229°C; crystalized from ethanol; IR (KBr, υ cm^{-1}): 1736 (C=O), 1620 (2C=N); ^{1}H NMR (DMSO-d6): δ 2.07 (s, 3H, CH_3), 6.76 (s, 1H, CH-thiazole proton), 7.07-7.13 (m, 1H, Ar-H), 7.19-7.22 (m, 1H, Ar-H), 7.28-7.33 (m, 2H, Ar-H), 7.38-7.41 (m, 2H, Ar-H), 7.45-7.50 (m, 3H, Ar-H), 7.58 (s, 1H, Ar-H), 7.75-7.79 (m, 1H, Ar-H), 8.11 (d, 1H, Ar-H, J=7.2 Hz,), 8.14 (s, 1H, Ar-H); MS (EI) m/z (%): 593.10, 595.02, 597.01 (M+, 49, 100, 50); Anal. Calcd. for $C_{26}H_{17}Br_2N_3O_2S$ (595.31): C, 52.46; H, 2.88; N, 7.06; S, 5.39; Found: C, 52.72; H, 3.07; N, 7.23; S, 5.52.

2-(2-((1-(6-Bromo-2-oxo-2H-chromen-3-yl)ethylidene)hydrazono)-3-methyl/phenyl-4-oxothiazolidin-5-yl)acetic acid **6a,b**

To a hot solution of compound 2a, b (1.2 mmol) in toluene (20 mL), maleic anhydride (1.2 mmol) was added. The reaction mixture was refluxed for 16h till reaction completion, then left to cool. The formed precipitate was filtered, dried and recrystallized from the proper solvent to afford the corresponding title compounds **6a,b**.

2-(2-((1-(6-Bromo-2-oxo-2H-chromen-3-yl)ethylidene)hydrazono)-3-methyl-4-oxothiazolidin-5-yl)acetic acid (**6a**)

Yield 83%; as buff powder; m.p. 280°C; crystalized from ethanol; IR (KBr, υ cm^{-1}): 3441 (OH), 1721, 1675 (2C=O), 1597 (2C=N); ^{1}H NMR (DMSO-d6): δ 2.35 (s, 3H, CH_3), 2.87-2.95 (m, 1H, CH_2), 3.04-3.09 (m, 1H, CH_2), 3.20 (s, 3H, CH_3), 4.39 (t, 1H, CH), 7.42 (d, 1H, Ar-H, J=8.0 Hz,), 7.80 (d, 1H, Ar-H, J=6.4 Hz,), 8.17 (s, 2H, Ar-H), 12.74 (s, 1H, OH, D_2O exchangeable); ^{13}C NMR (DMSO-d6): δ 17.28 (CH_3), 29.99 (NCH_3), 37.23 (CH_2), 43.21 (CH), 116.77, 118.74, 121.10, 127.90, 131.82, 135.31, 140.77, 153.04, 159.00, 160.96, 164.55, 172.12, , 174.44; MS (EI) m/z (%): 451, 453 (M+, 11, 13), 55 (C4H7, 100) ; Anal. Calcd. for $C_{17}H_{14}BrN_3O_5S$ (452.28): C, 45.15; H, 3.12; N, 9.29; S, 7.09; Found: C, 45.38; H, 3.29; N, 9.48, S, 6.78.

2-(2-((1-(6-Bromo-2-oxo-2H-chromen-3-yl)ethylidene)hydrazono)-4-oxo-3-phenylthiazolidin-5-yl)acetic acid (**6b**)

Yield 50%; buff powder; m.p. 251°C crystalized from glacial acetic acid; IR (KBr, υ cm^{-1}): 3449 (OH), 1721, 1605 (2C=O), 1566 (2C=N); ^{1}H NMR (DMSO-d6): δ 2.09 (s, 3H, CH_3), 3.12 (d, 2H, CH_2, J=4 Hz,), 4.58 (t, 1H, CH, J=8 Hz,), 7.40-7.48 (m, 4H, Ar-H), 7.52-7.56 (m, 2H, Ar-H), 7.80 (dd, 1H, Ar-H, J=4.0, 8.0 Hz,), 8.17 (s, 2H, Ar-H); ^{13}C NMR (DMSO-d6) : δ 17.23 (CH_3), 37.24 (CH_2), 43.14 (CH), 116.77, 118.74, 121.06, 127.71, 128.39, 129.08, 129.39, 131.81, 135.35, 135.64, 140.87, 153.03, 158.91, 161.13 (Ar-C), 164.41 (C=O), 172.16 (C=N), 172.51, 174.10; MS (EI) m/z (%): 513, 515 (M+, 100, 85); Anal. Calcd. for $C_{22}H_{16}BrN_3O_5S$ (514.35): C, 51.37; H, 3.14; N, 8.17; S, 6.23; Found: C, 51.60; H, 3.26; N, 8.41; S, 6.02.

General procedure for synthesis of 1-(substituted) thiosemicarbazides (Schiff bases) **7a–i**

The synthesis of thiosemicarbazones was carried out according to the reported procedures57-63 and the brief procedure is provided in (Supplementary material).

General procedure for the synthesis of 3-(2-(2-(substituted)hydrazinyl)thiazol-4-yl)-6-bromo-2H-chromen-2-one **9a-i**

Supporting information

6-Bromo-3-(2-bromoacetyl)-2H-chromen-2-one (0.69 g, 0.002 mol) and the appropriate thiosemicarbazone (0.002 mol) were dissolved in ethanol (10 ml). The reaction mixture was refluxed for 5-7 h, monitored by TLC. The formed precipitate was filtered while hot, dried and recrystallized from the proper solvent to afford the corresponding title compounds 9a-i respectively.

3-(2-(2-Benzylidenehydrazinyl)thiazol-4-yl)-6-bromo-2H-chromen-2-one (**9a**)

Yield 75%; pale green powder; m.p. 234°C; crystallized from DMF and ethanol; IR (νmax/cm^{-1}): 3456 (NH), 1705 (C=O),1635 (C=N), 1574 (C=C); ^{1}H NMR (DMSO-d6): δ 7.37-7.51 (m, 4H, Ar-H), 7.65 (d, 2H, Ar-H, J=7.2 Hz), 7.75 (dd, 1H, Ar-H, J=3.2, 9.2 Hz), 7.80 (s, 1H, CH), 8.01 (s, 1H, Ar-H), 8.12 (s, 1H, Ar-H), 8.47 (s, 1H, Ar-H), 12.23 (s, 1H, NH, D_2O exchangeable); ^{13}C NMR (DMSO-d6) : δ 111.99, 116.84, 118.58, 121.61, 121.89, 126.78, 129.32, 131.12, 134.36, 134.70, 137.18, 142.32, 144.19, 151.75, 158.76, 168.23; MS (EI) m/z (%): 426, 428 (M+1 , 51, 49), 346 ($C_{13}H_5BrN_3O_2S$, 100, 38); Anal. Calcd. for $C_{19}H_{12}BrN_3O_2S$ (426.29): C, 53.53; H, 2.84; N, 9.86; S, 7.52; Found: C, 53.78; H, 3.07; N, 9.97; S, 7.24.

6-Bromo-3-(2-(2-(4-methylbenzylidene)hydrazinyl) thiazol-4-yl)-2H-chromen-2-one (**9b**)

Yield 71%; yellow powder; m.p. 250°C; crystallized from glacial acetic acid; IR (KBr, υ cm-1): 3472 (NH), 1636 (C=O of coumarin), 1520 (C=N of thiazole), 1474 (C=N); ^{1}H NMR (DMSO-d6): δ 2.33 (s, 3H, CH3), 7.22 (d, 2H, Ar-H, J = 6.4 Hz), 7.39-7.45 (m, 1H, Ar-H), 7.53 (d, 2H, Ar-H, J = 9.6 Hz), 7.72-7.83 (m, 2H, Ar-H), 8.03 (s, 1H, Ar-H), 8.11 (s, 1H, Ar-H), 8.44 (s, 1H, Ar-H), 12.11 (s, 1H, NH, D_2O exchangeable); ^{13}C NMR (DMSO-d6): δ 21.55 (CH_3), 111.91, 116.94, 119.07, 121.66, 127.01, 129.91, 131.82, 132.09, 134.27, 135.31, 137.04, 139.94, 142.52, 143.43, 144.25, 151.73, 153.00, 159.08, 168.29; MS (EI) m/z (%): 439, 441 (M+ , 49, 69), 286 ($C_{14}H_{12}N_3O_2S$, 100); Anal. Calcd. for $C_{20}H_{14}BrN_3O_2S$ (440.32): C, 54.56; H, 3.20; N, 9.54; S, 7.28; Found: C, 54.79; H, 3.41; N, 9.80; S, 7.04.

6-Bromo-3-(2-(2-(3,4-dimethoxybenzylidene)hydrazinyl)thiazol-4-yl)-2H-chromen-2-one (**9c**)

Yield 69%; pale green powder; m.p. 238°C; crystallized from glacial acetic acid; IR (KBr, υ cm^{-1}): 3433 (NH), 1728 (C=O of coumarin), 1520 (C=N of thiazole), 1474 (C=N); ^{1}H NMR (DMSO-d6): δ ppm: 3.80 (s, 3H, OCH_3), 3.82 (s, 3H, OCH_3), 7.01 (d, 1H, Ar-H, J=8.0 Hz), 7.18 (d, 1H, Ar-H J=12.0 Hz,), 7.29-7.44 (m, 3H, Ar-H), 7.79 (s, 1H, CH), 8.00 (s, 1H, Ar-H), 8.13 (s, 1H, Ar-H), 8.47 (s, 1H, Ar-H), 12.13 (s, 1H, NH, D_2O exchangeable); ^{13}C NMR (DMSO-d6): δ 55.84 (OCH_3), 56.03 (OCH_3), 107.16, 111.09, 116.85, 117.78, 120.93, 121.62, 121.92, 126.32, 127.48, 131.09, 134.63, 137.17, 142.60, 145.41, 150.67, 151.74, 152.94, 158.81, 168.30; MS (EI) m/z (%): 486, 488 (M+1 , 24, 10), 152 ($C_9H_{12}O_2$, 100); Anal. Calcd. for $C_{21}H_{16}BrN_3O_4S$ (486.34): C, 51.86; H, 3.32; N, 8.64; S, 6.59; Found: C, 52.05; H, 3.47; N, 8.91; S, 6.41.

6-Bromo-3-(2-(2-(4-nitrobenzylidene)hydrazinyl)thiazol-4-yl)-2H-chromen-2-one (**9d**)

yield 74%; orange powder; m.p. 276°C; crystallized from glacial acetic acid; IR (KBr, υ cm^{-1}): 3367 (NH), 1705 (C=O), 1635 (C=C), 1381

(NO_2); ^1H NMR (DMSO-d6) δ : 7.37 (d, 1H, Ar-H, J=6.8 Hz), 7.70 (dd, 1H, Ar-H, J=3.6, 8.8 Hz,), 7.83-7.87 (m, 3H, Ar-H), 8.09 (d, 1H, Ar-H, J=3.6 Hz), 8.12 (s, 1H, CH), 8.22 (d, 2H, Ar-H, J=10 Hz), 8.42 (s, 1H, Ar-H), 12.62 (s, 1H, NH, D_2O exchangeable); ^{13}C NMR (DMSO-d6): δ 111.31, 112.64, 116.85, 118.56, 121.51, 121.75, 124.34, 124.54, 127.45, 131.10, 134.39, 137.22, 139.51, 141.09, 144.27, 147.59, 151.73, 158.69, 167.73; MS (EI) m/z (%): 471, 473 (M+1, 36, 17), 114 ($C_3H_4N_3S$, 100); Anal. Calcd. for $C_{19}H_{11}BrN_4O_4S$ (471.29): C, 48.42; H, 2.35; N, 11.89; S, 6.80; Found: C, 48.70; H, 2.51; N, 12.13; S, 7.01.

6-Bromo-3-(2-(2-(4-(dimethylamino)benzylidene)hydrazinyl)thiazol-4-yl)-2H-chromen-2-one (**9e**)

Yield 85%; orange powder; m.p. 253°C; crystallized from glacial acetic acid ; IR (KBr, υ cm-1): 3372 (NH), 1697 (C=O), 1636 (C=C); ^1H NMR (DMSO-d6) δ : 3.00 (s, 6H, $N(CH_3)2$), 6.88-6.94 (d, 2H, Ar-H, J= 10.8 Hz), 7.37 (d, 1H, Ar-H, J= 12.2 Hz), 7.51 (d, 2H, Ar-H, J= 12.2 Hz), 7.71-7.74 (m, 2H, Ar-H), 7.97 (s, 1H, CH), 8.09 (s, 1H, Ar-H), 8.44 (s, 1H, Ar-H), 11.96 (s, 1H, NH, D_2O exchangeable); ^{13}C NMR (DMSO-d6): δ 33.01 ($2CH_3$), 111.48, 113.55, 116.83, 118.58, 121.66, 121.95, 128.13, 131.10, 134.32, 137.08,, 143.12, 144.11, 151.74, 153.00, 158.79, 168.35; MS (EI) m/z (%): 468, 470 (M+ , 67, 55), 234 (C11H14N4S, 100); Anal. Calcd. for: $C_{21}H_{17}BrN_4O_2S$ (469.36): C, 53.74; H, 3.65; N, 11.94; S, 6.83; Found: C, 53.61; H, 3.84; N, 12.07; S, 6.51.

6-Bromo-3-(2-(2-(4-fluorobenzylidene)hydrazinyl)thiazol-4-yl)-2H-chromen-2-one (**9f**)

Yield 58%; canary yellow powder; m.p. 278 °C; crystallized from DMF and ethanol; IR (KBr, υ cm^{-1}): 3464 (NH), 1728 (C=O), 1635 (C=C); ^1H NMR (DMSO-d6) δ :7.24-7.33 (m, 2H, Ar-H), 7.38-7.51 (m, 2H, Ar-H), 7.69-7.73 (m, 1H, Ar-H), 7.78 (d, J=2.8 Hz, 1H, Ar-H), 8.04 (s, 1H, CH), 8.09 (s, 1H, Ar-H), 8.26 (s, 1H, Ar-H), 8.45 (s, 1H, Ar-H), 12.30 (s, 1H, NH, D_2O exchangeable); ^{13}C NMR (DMSO-d6): δ 115.96, 116.18, 116.94, 117.20, 118.87, 119.97, 120.55, 121.90, 130.80, 132.04, 132.79, 133.54, 138.12, 142.87, 148.79, 153.09, 154.92, 165.52, 173.38; MS (EI) m/z (%): 442, 444, 445 (M-1 , 14, 53, 12), 385, 387 ($C_{16}H_8BrN_3O_2S$, 53, 100); Anal. Calcd.for $C_{19}H_{11}BrFN_3O_2S$ (444.28): C, 51.37; H, 2.50; N, 9.46; S, 7.22; Found: C, 51.59, H, 2.79; N, 9.59; S, 7.01.

6-Bromo-3-(2-(2-(4-bromobenzylidene)hydrazinyl)thiazol-4-yl)-2H-chromen-2-one (**9g**)

Yield 93%; dark yellow powder; m.p. 267°C; crystallized from DMF and ethanol; IR (νmax/cm^{-1}): 3418 (NH), 1705 (C=O), 1635 (C=C); ^1H NMR (DMSO-d6) δ : 7.38 (d, 1H, Ar-H, J=9.6 Hz,), 7.57-7.64 (m, 4H, Ar-H), 7.73 (dd, J=3.6, 7.2Hz, 1H, Ar-H), 7.79 (s, 1H, CH), 8.02 (s, 1H, Ar-H), 8.09 (s, 1H, Ar-H), 8.43 (s, 1H, Ar-H), 12.31 (s, 1H, NH, D_2O exchangeable); ^{13}C NMR (DMSO-d6): δ 112.12, 116.84, 118.58, 119.24, 121.60, 121.86, 122.93, 128.60, 131.13, 132.28, 134.02, 134.36, 137.20, 141.01, 144.21, 151.75, 158.75,, 168.08, 175.20; MS (EI) m/z (%): 502, 504, 506 (M+,42, 68, 61), 199 ($C_{10}H_5N_3S$, 100); Anal. Calcd. for: $C_{19}H_{11}Br_2N_3O_2S$ (505.18): C, 45.17; H, 2.19; N, 8.32; S, 6.35; Found: C, 45.33; H,2.45; N, 8.59; S, 6.11.

6-Bromo-3-(2-(2-((5-methylfuran-2-yl)methylene)hydrazinyl)thiazol-4-yl)-2H-chromen-2-one (**9h**)

Supporting information

Yield 67%; dark green powder; m.p. 268°C; crystallized from DMF and ethanol ; IR (KBr, υ cm^{-1}): 3441 (NH), 1690 (C=O), 1620 (C=C); ^{1}H NMR (DMSO-d6) δ : 2.37 (s, 3H, CH_3), 6.23 (d, 1H, Ar-H, J=4.8 Hz,), 6.71 (d, 1H, Ar-H, J=2.8 Hz), 7.41 (d, 1H, Ar-H, J=10.0 Hz), 7.69 (d, J=1H, Ar-H, 5.6 Hz,), 7.78 (s, 1H, CH), 7.90 (s, 1H, Ar-H), 8.14 (s, 1H, Ar-H), 8.46 (s, 1H, Ar-H), 12.10 (s, 1H, NH, D_2O exchangeable); ^{13}C NMR (DMSO-d6): δ 14.17 (CH_3), 106.27, 108.98, 111.90, 116.90, 118.60, 121.34, 122.09, 133.00, 140.30, 140.40, 140.48, 152.09, 166.26 (Ar-C), 134.00 (CH), 169.57 (C=O), 171.23 (C=N); MS (EI) m/z (%): 430, 432 (M+ , 100, 85); Anal. Calcd. for: $C_{18}H_{12}BrN_3O_3S$ (430.28): C, 50.25; H, 2.81; N, 9.77; S, 7.45; Found: C, 50.53; H, 3.04; N,9.94; S, 7.14.

6-Bromo-3-(2-(2-((5-nitrofuran-2-yl)methylene)hydrazinyl)thiazol-4-yl)-2H-chromen-2-one (**9i**)

Yield 82%; orange powder; m.p. 274°C; crystallized from DMF and ethanol; IR (KBr, υ cm^{-1}): 3418 (NH), 1636 (C=O of coumarin), 1566 (NO_2), 1520 (C=N of thiazole), 1489 (C=N), 1381 (NO_2); ^{1}H NMR (DMSO-d6) δ : 7.11 (d, 1H, Ar-H, J=6.0 Hz), 7.37 (d, 1H, Ar-H, J=10.4 Hz,), 7.72 (dd, 1H, Ar-H, J=2.8, 8.8 Hz,), 7.74 (d, 1H, Ar-H, J=4.0 Hz,), 7.83 (s, 1H, CH), 7.95 (s, 1H, Ar-H), 8.08 (s, 1H, Ar-H), 8.41 (s, 1H, Ar-H), 12.77 (s, 1H, NH, D_2O exchangeable); ^{13}C NMR (DMSO-d6): δ 110.03, 112.99, 115.52, 116.86, 118.58, 121.47, 124.96, 126.31, 129.79, 131.07, 138.16, 144.00, 152.39 (Ar-C), 134.47 (CH), , 158.67 (C=O), 168.00 (C=N); MS (EI) m/z (%): 460, 462 (M+1, 16, 33), 282 ($C_{14}H_8N_3O_2S$, 100); Anal. Calcd. for : $C_{17}H_9BrN_4O_5S$ (461.25): C, 44.27; H, 1.97; N, 12.15; S, 6.95; Found: C, 44.54; H, 2.18; N, 12.39; S, 7.31.

Supporting information

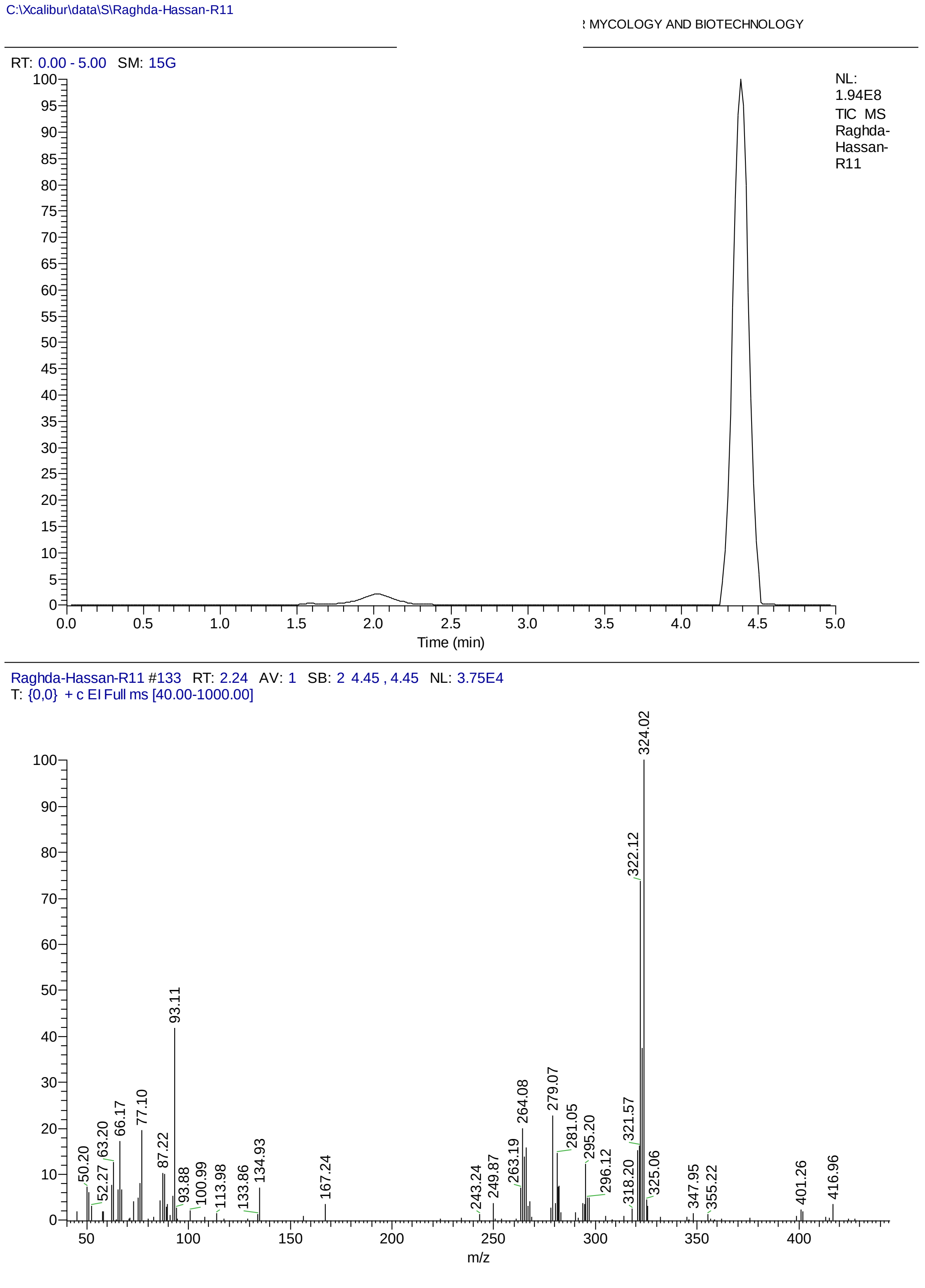

S1: Mass spectrum of compound 2b

Supporting information

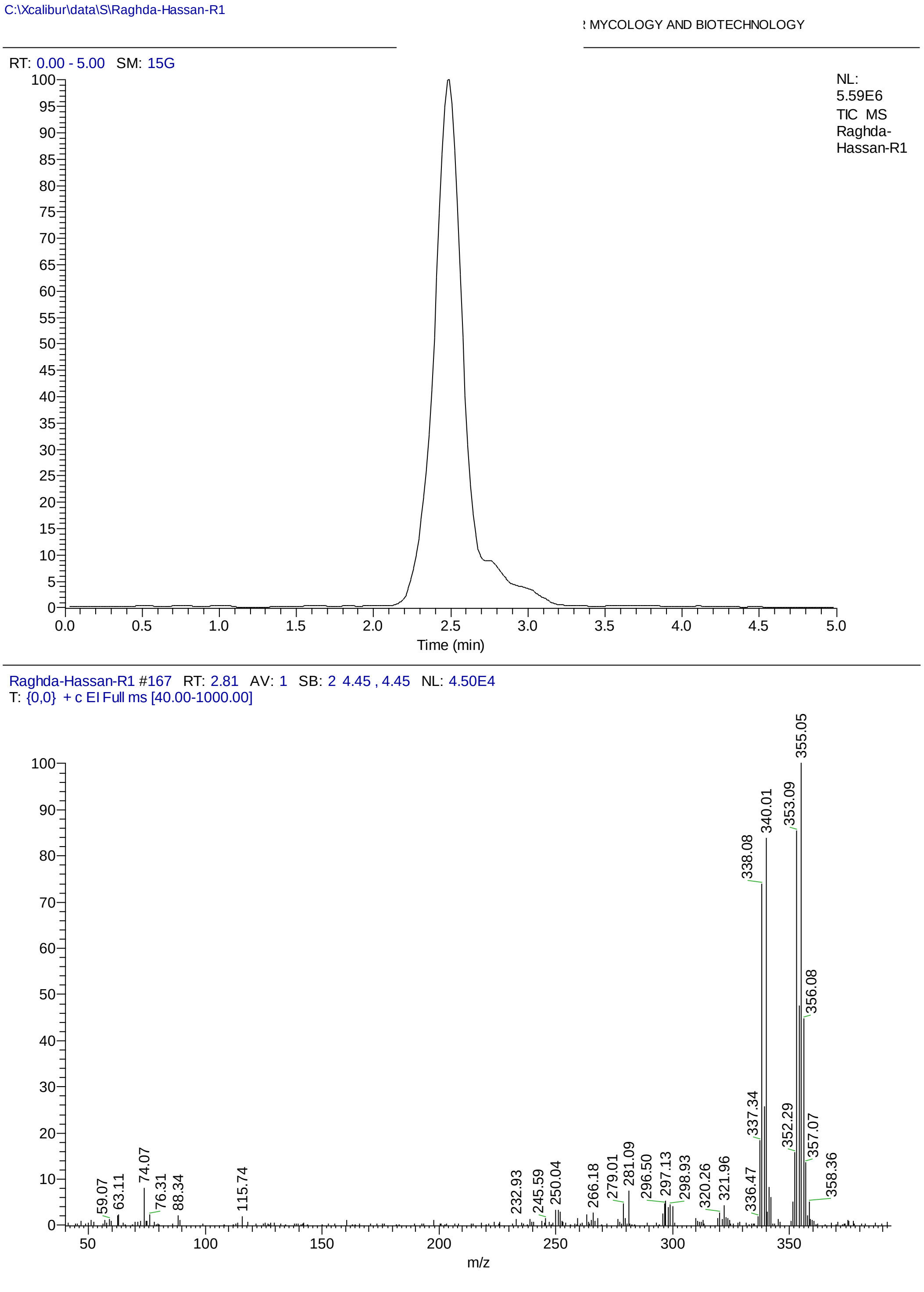

S2: Mass spectrum of compound 2a

Supporting information

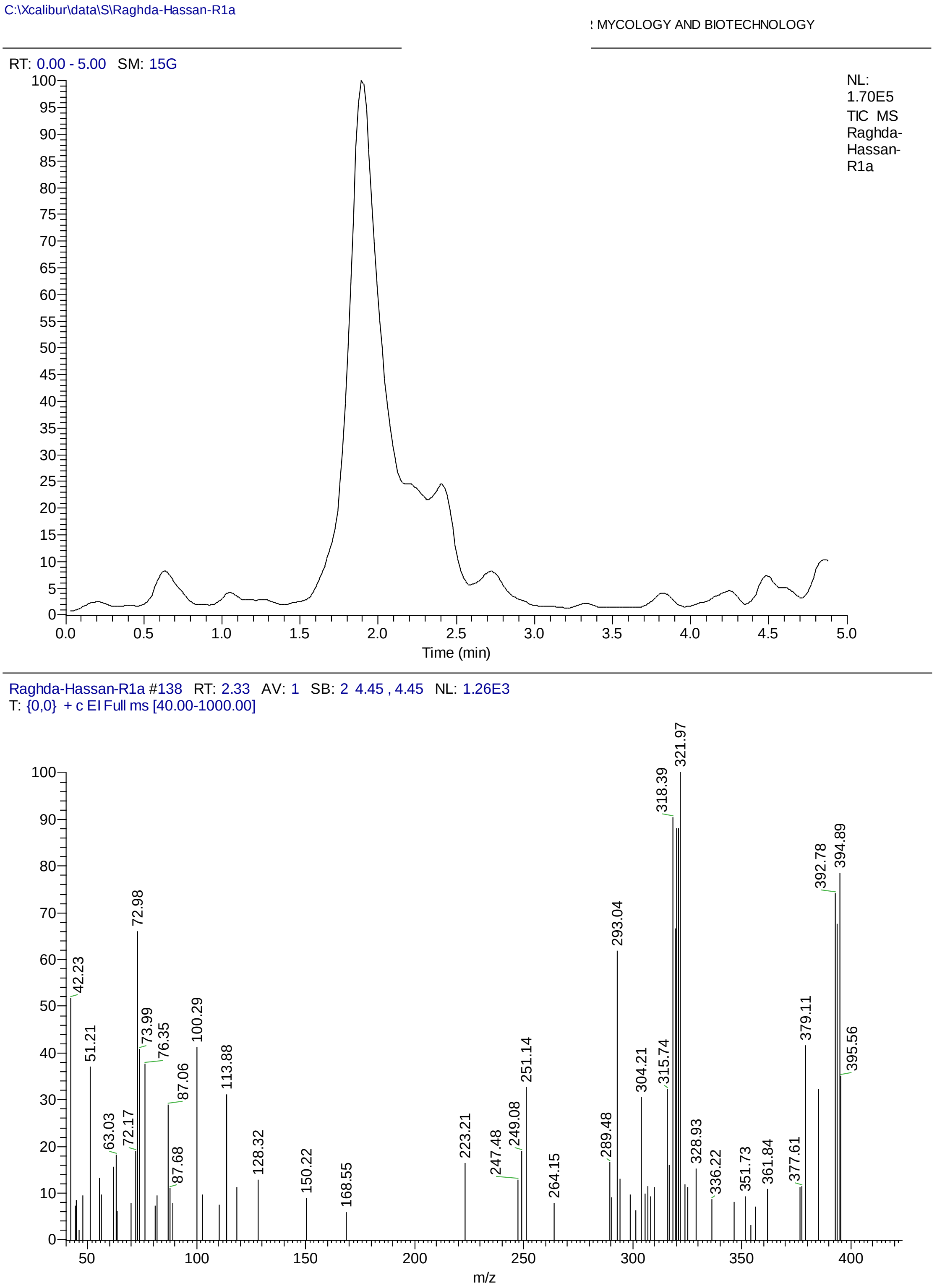

S3: Mass spectrum of compound 3a

Supporting information

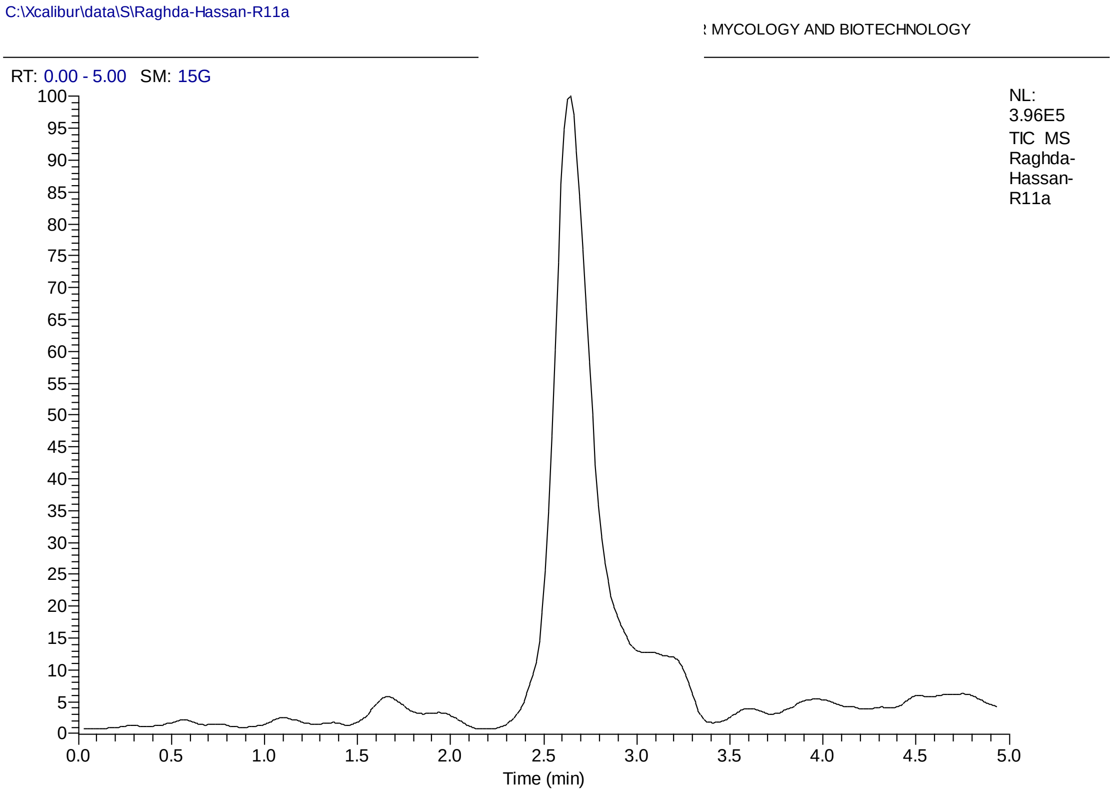

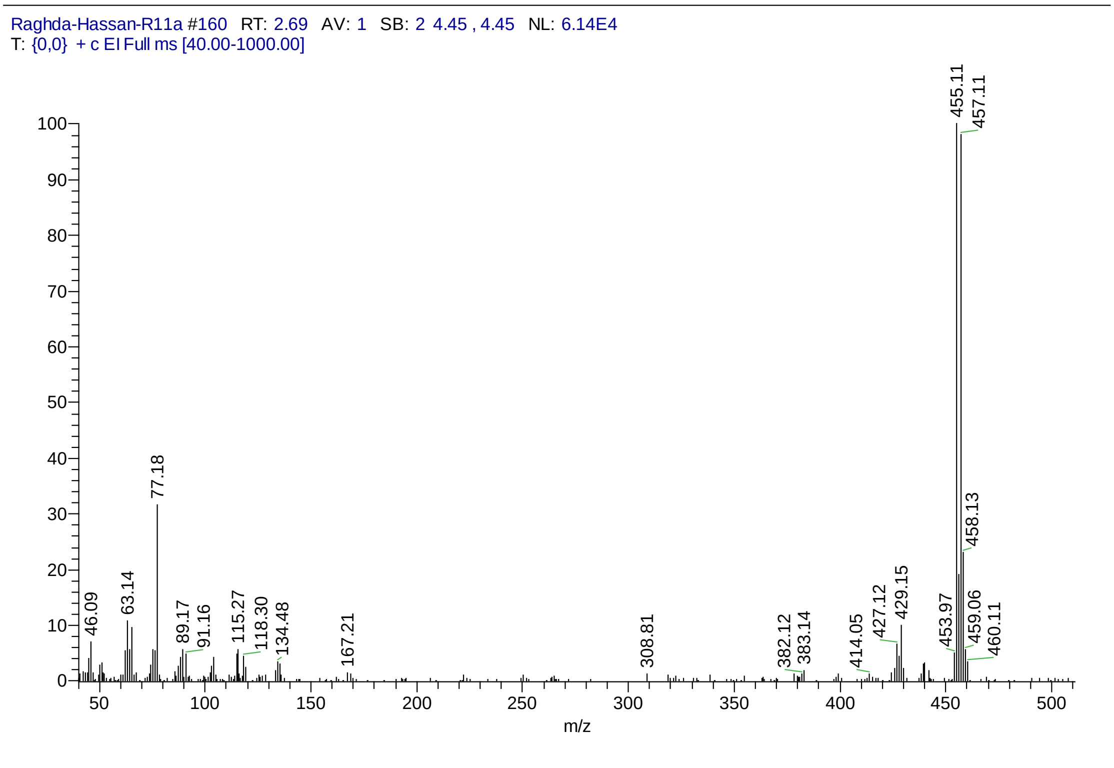

S4: Mass spectrum of compound 3b

Supporting information

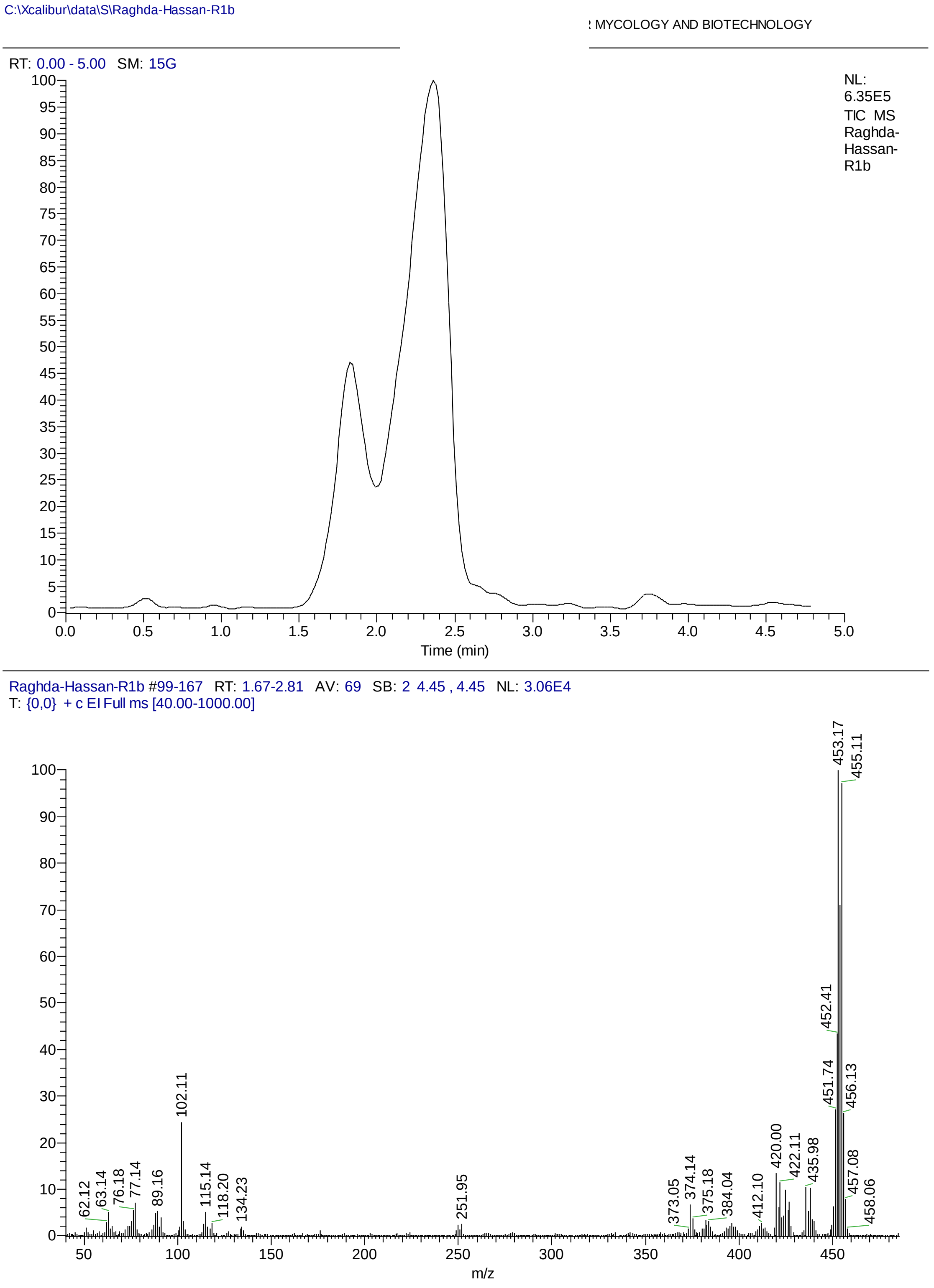

S5: Mass spectrum of compound 4a

Supporting information

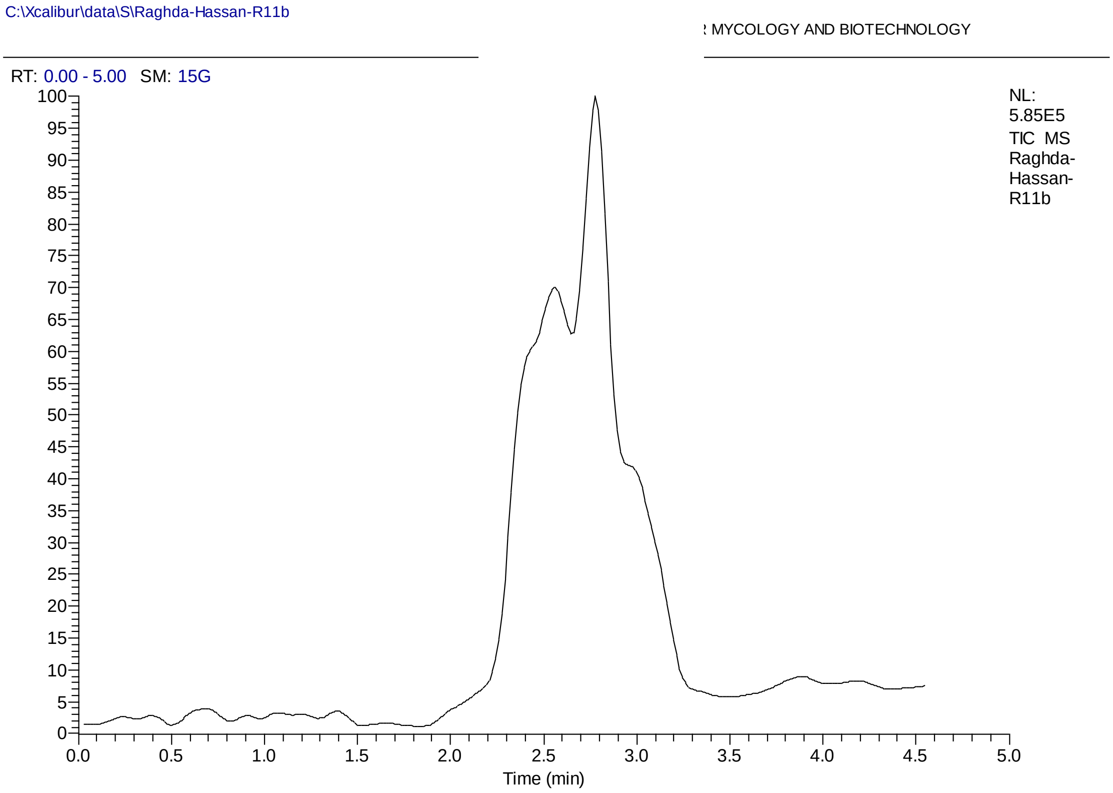

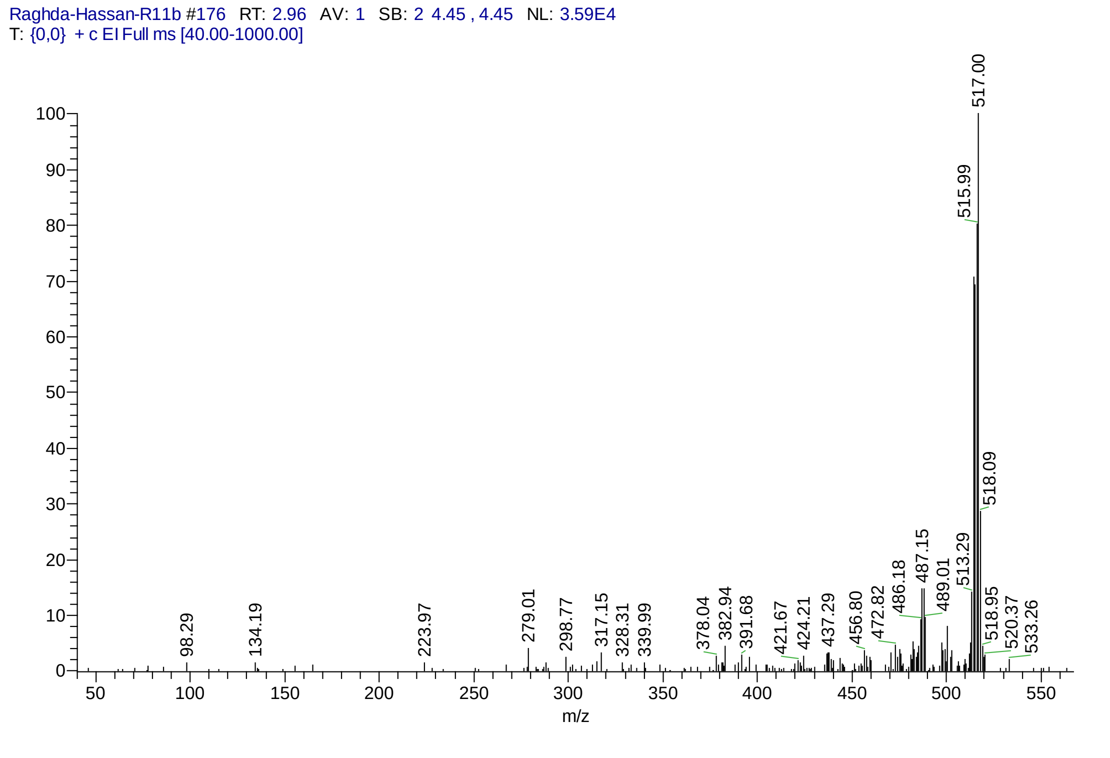

S6: Mass spectrum of compound 4b

Supporting information

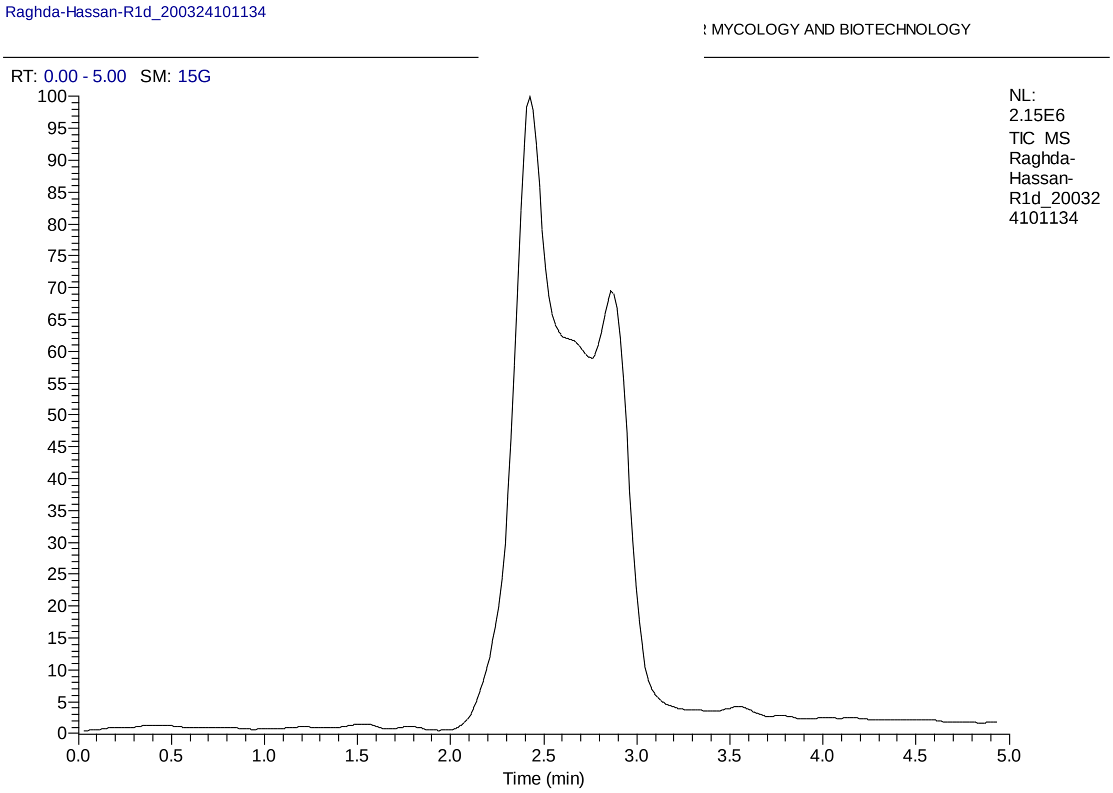

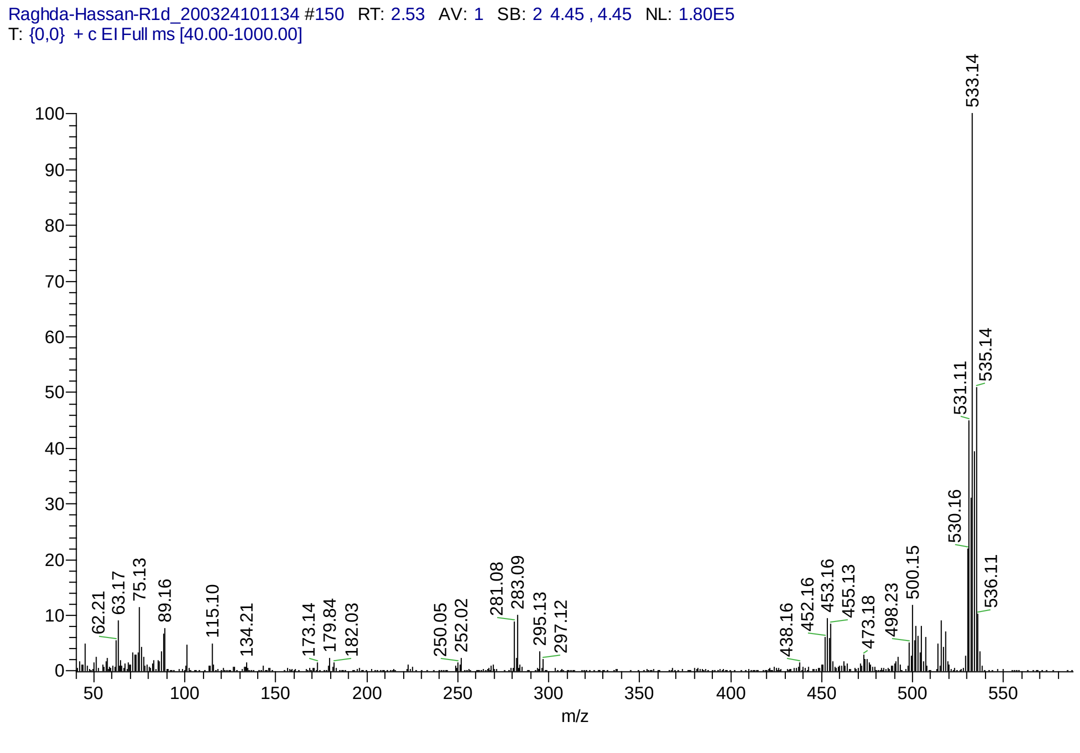

S7:Mass spectrum of compound 5a

Supporting information

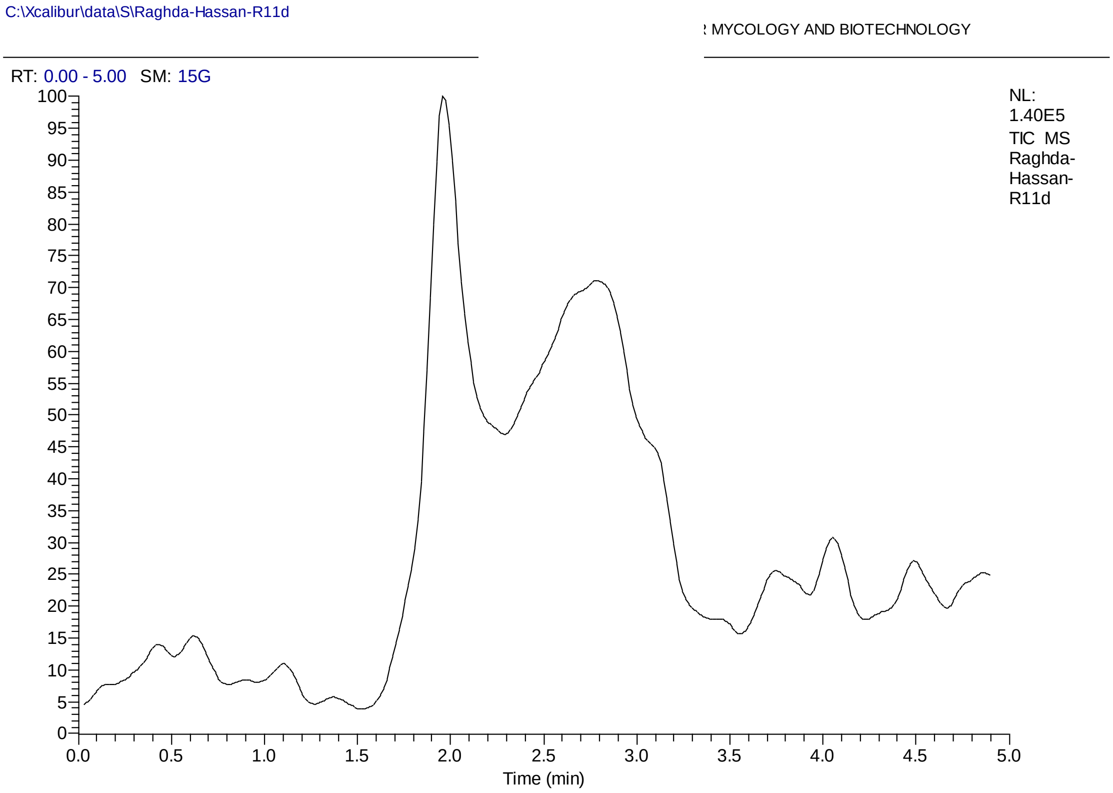

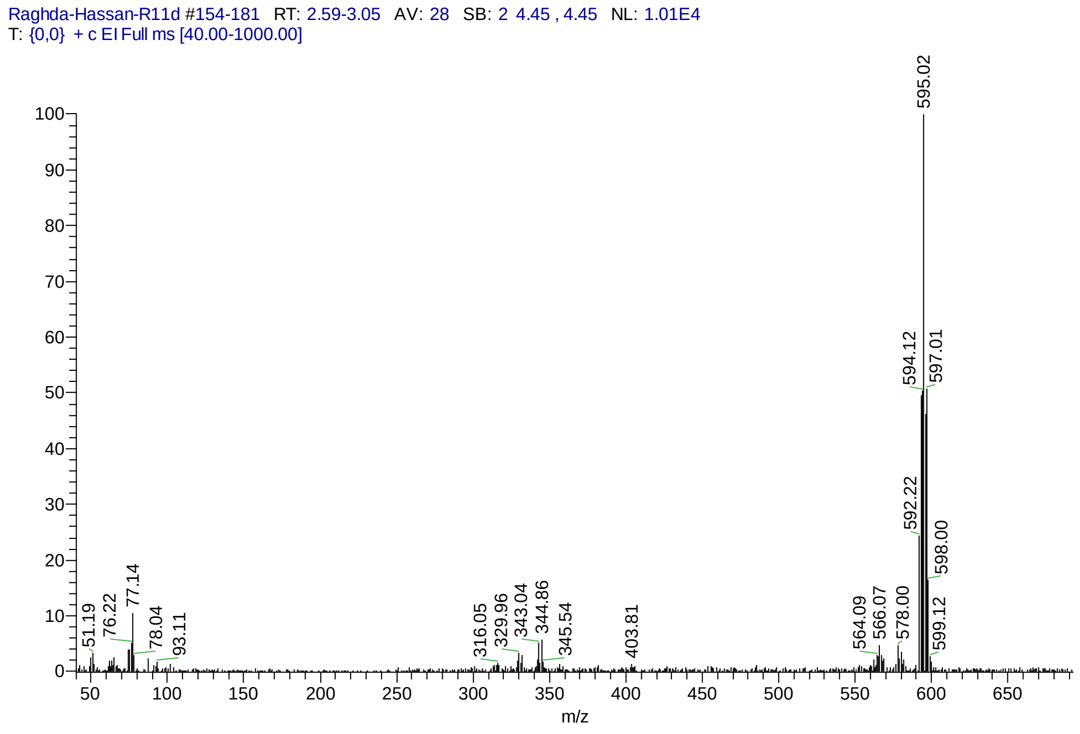

S8: Mass spectrum of compound 5b

Supporting information

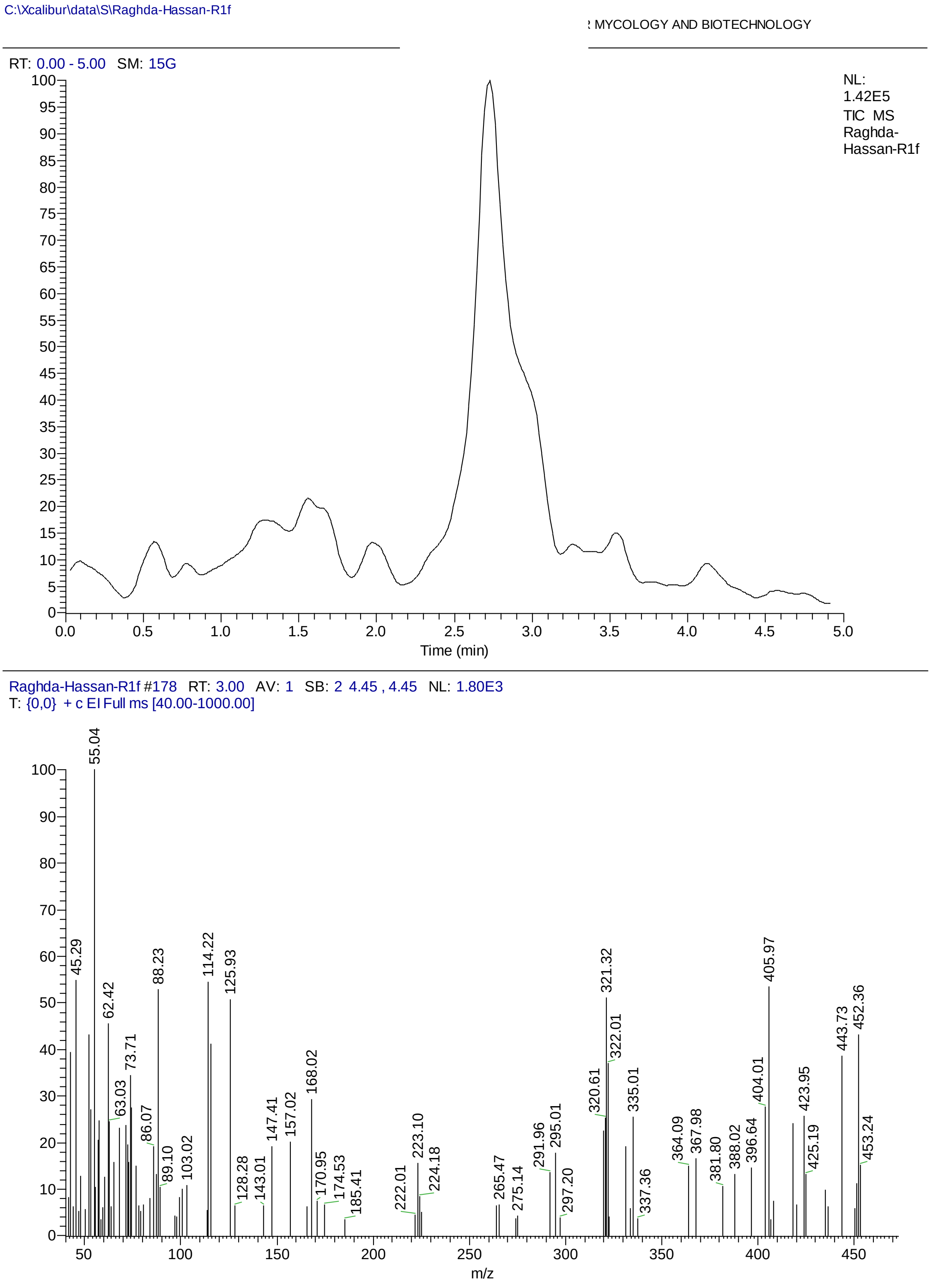

S9: Mass spectrum of compound 6a

Supporting information

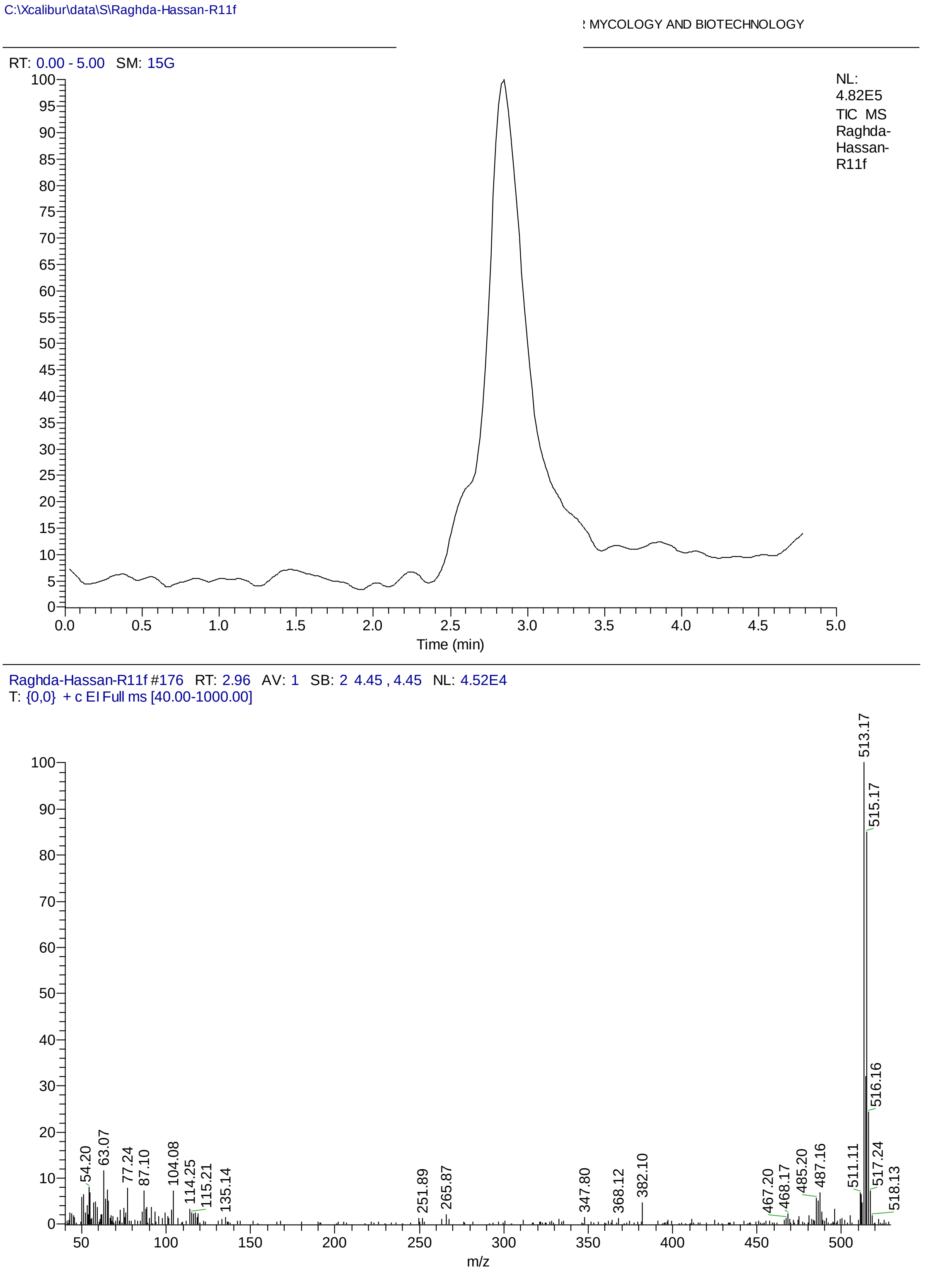

S10: Mass spectrum of compound 6b

Supporting information

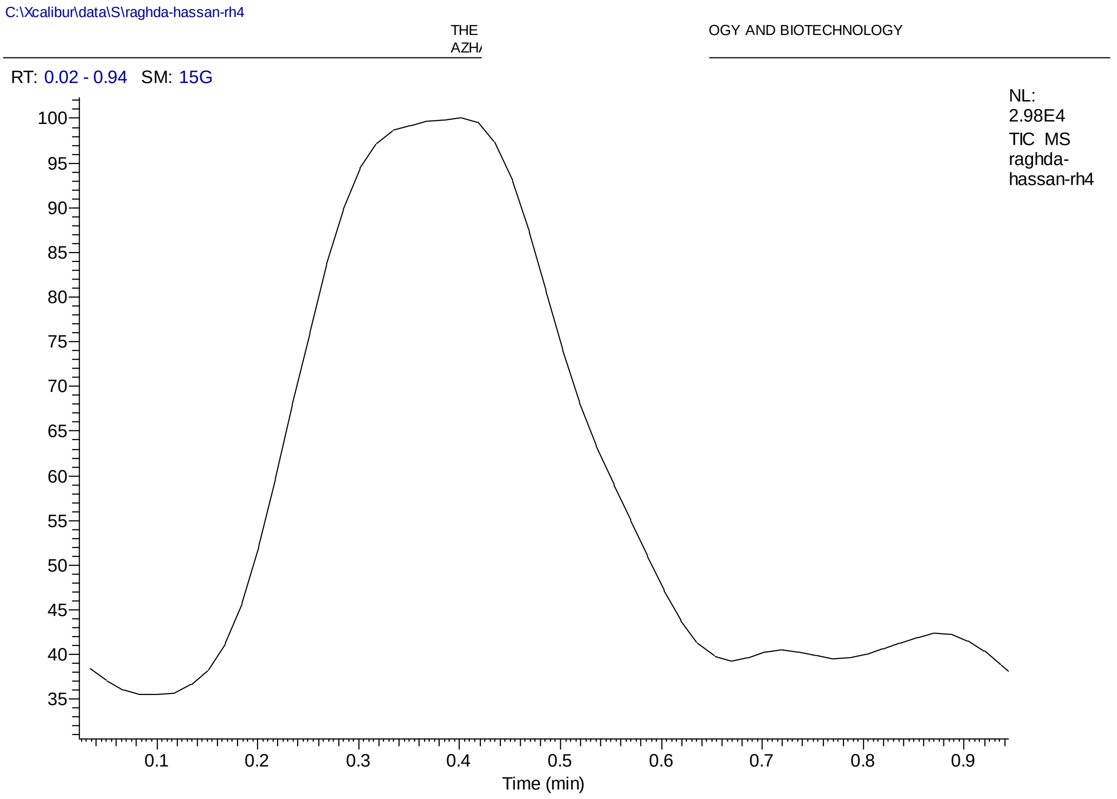

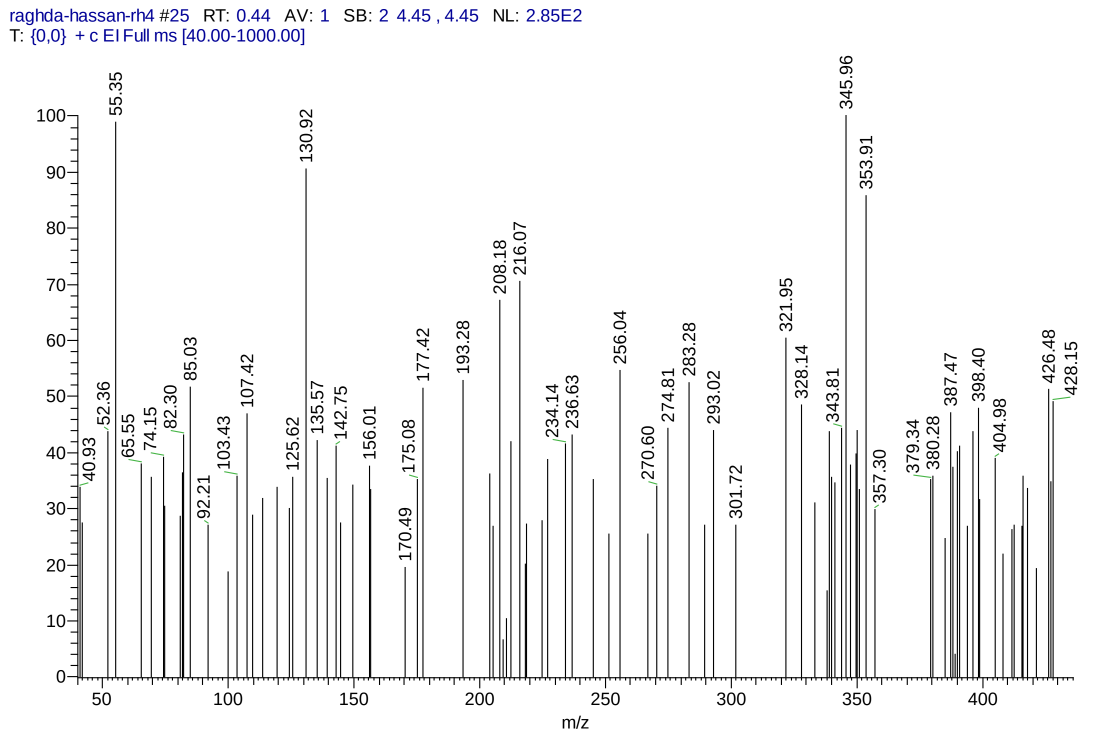

S11: Mass spectrum of compound 9a

Supporting information

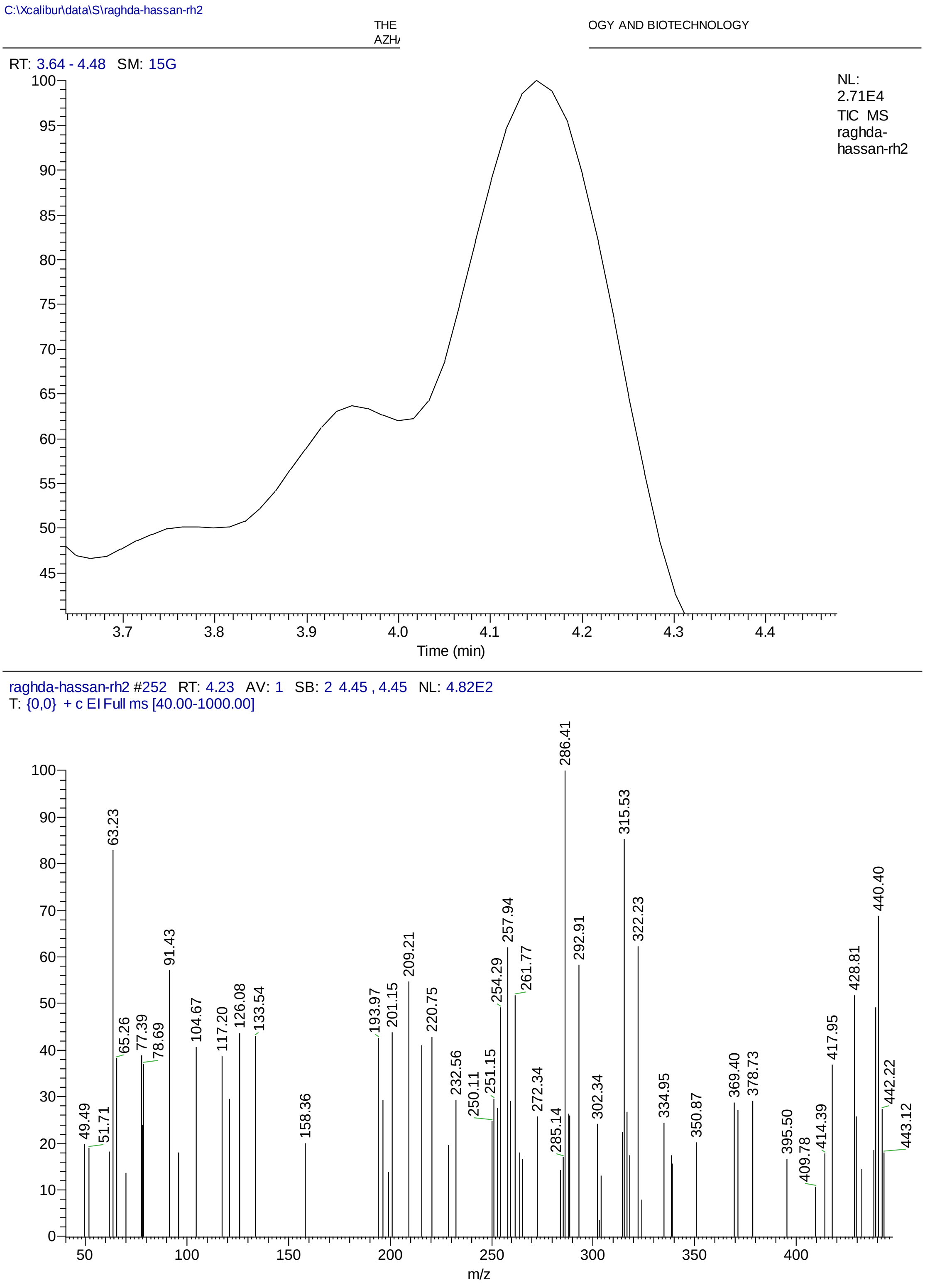

S12: Mass spectrum of compound 9b

Supporting information

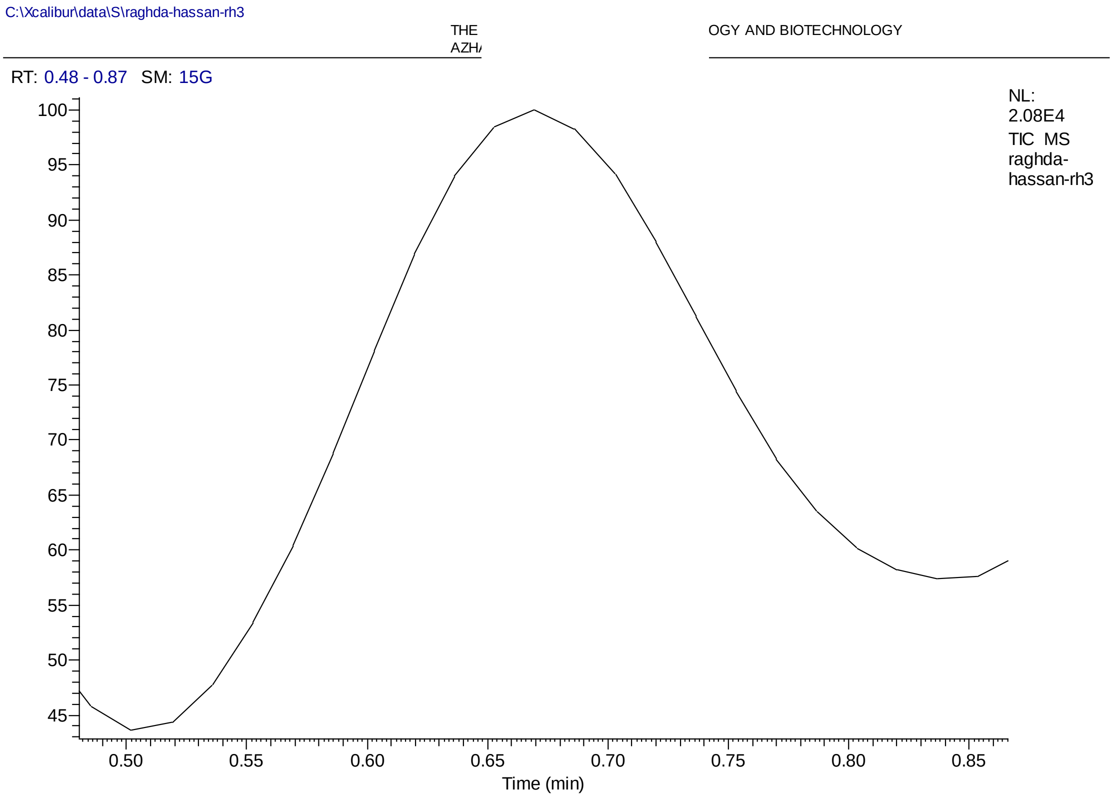

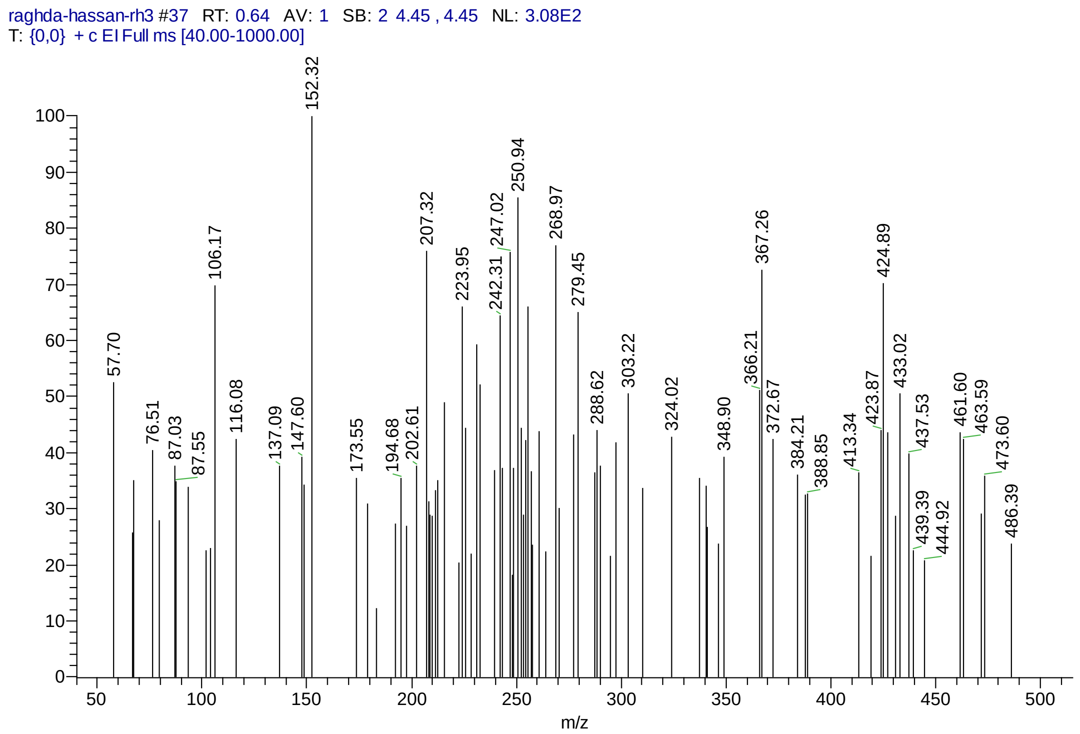

S13: Mass spectrum of compound 9c

Supporting information

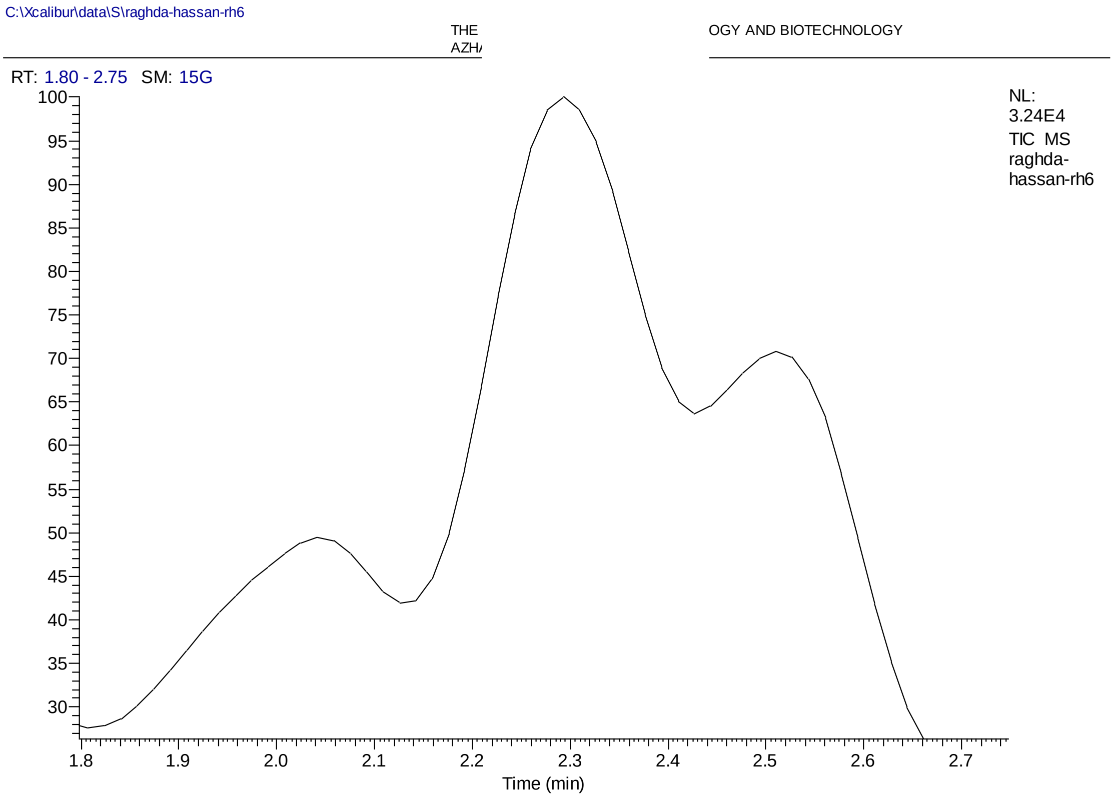

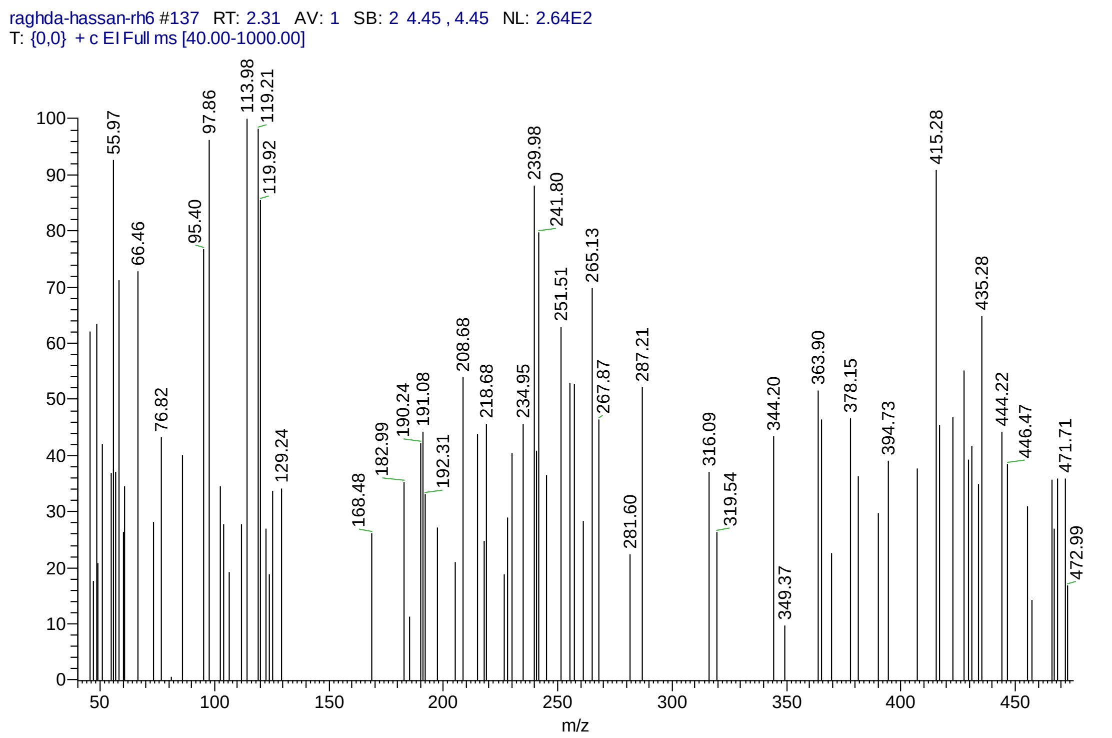

S14: Mass spectrum of compound 9d

Supporting information

C:\Xcalibur\data\S\raghda-hassan-rh8

THE AZH/ OGY AND BIOTECHNOLOGY

RT: 3.07 - 3.57 SM: 15G

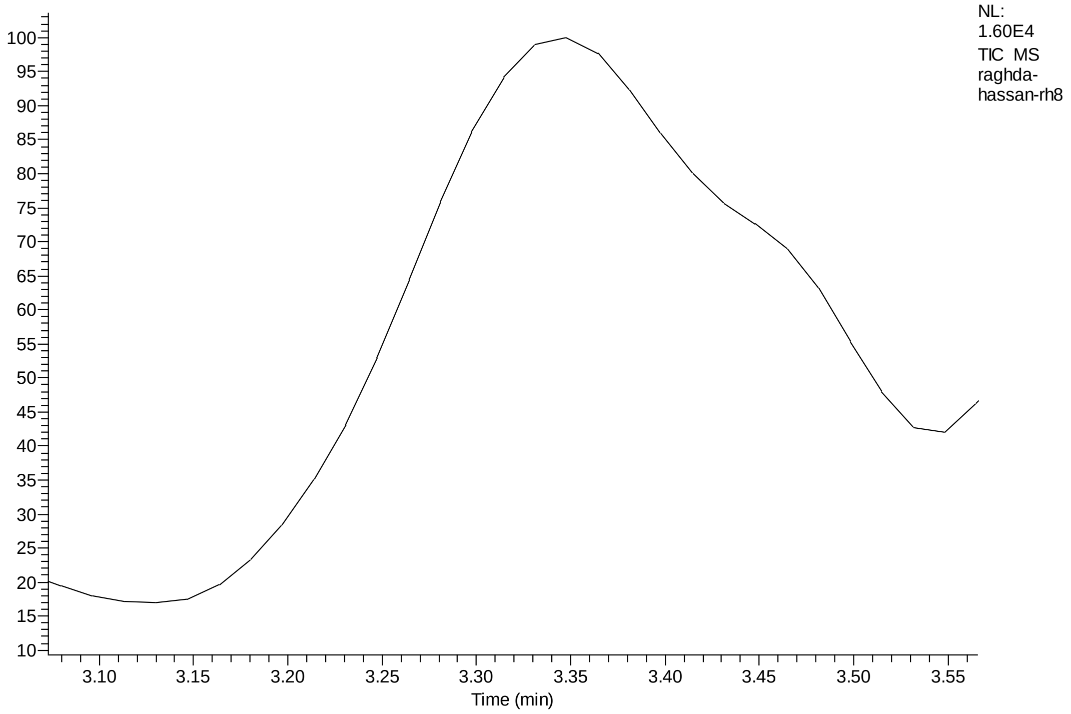

raghda-hassan-rh8 #196 RT: 3.30 AV: 1 SB: 2 4.45 , 4.45 NL: 4.39E2
T: {0,0} + c EI Full ms [40.00-1000.00]

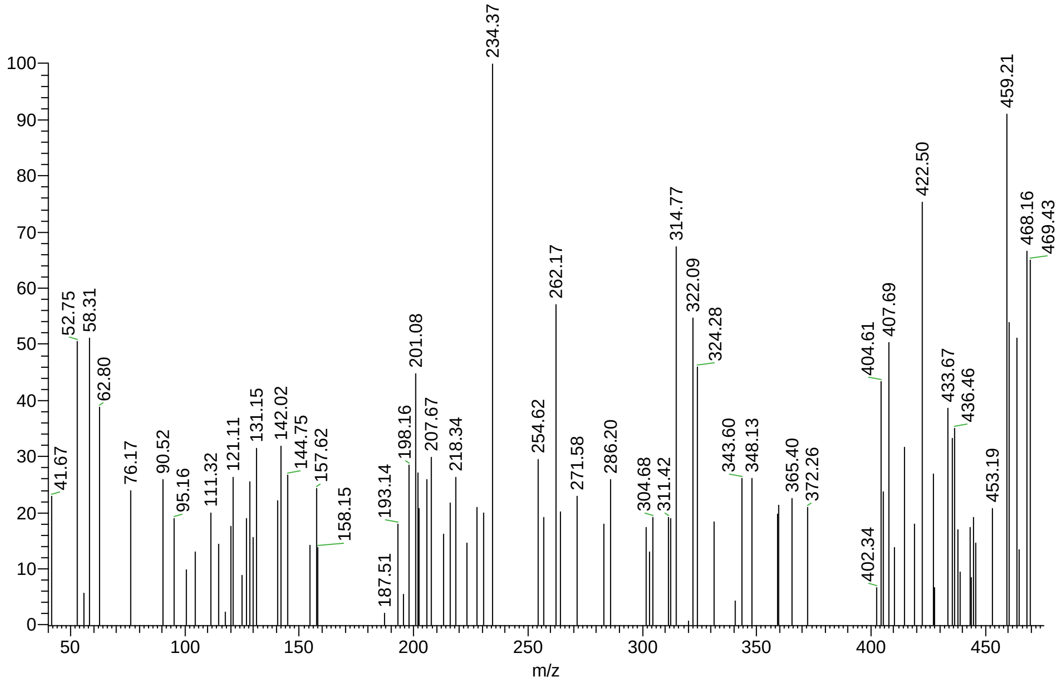

S15: Mass spectrum of compound 9e

Supporting information

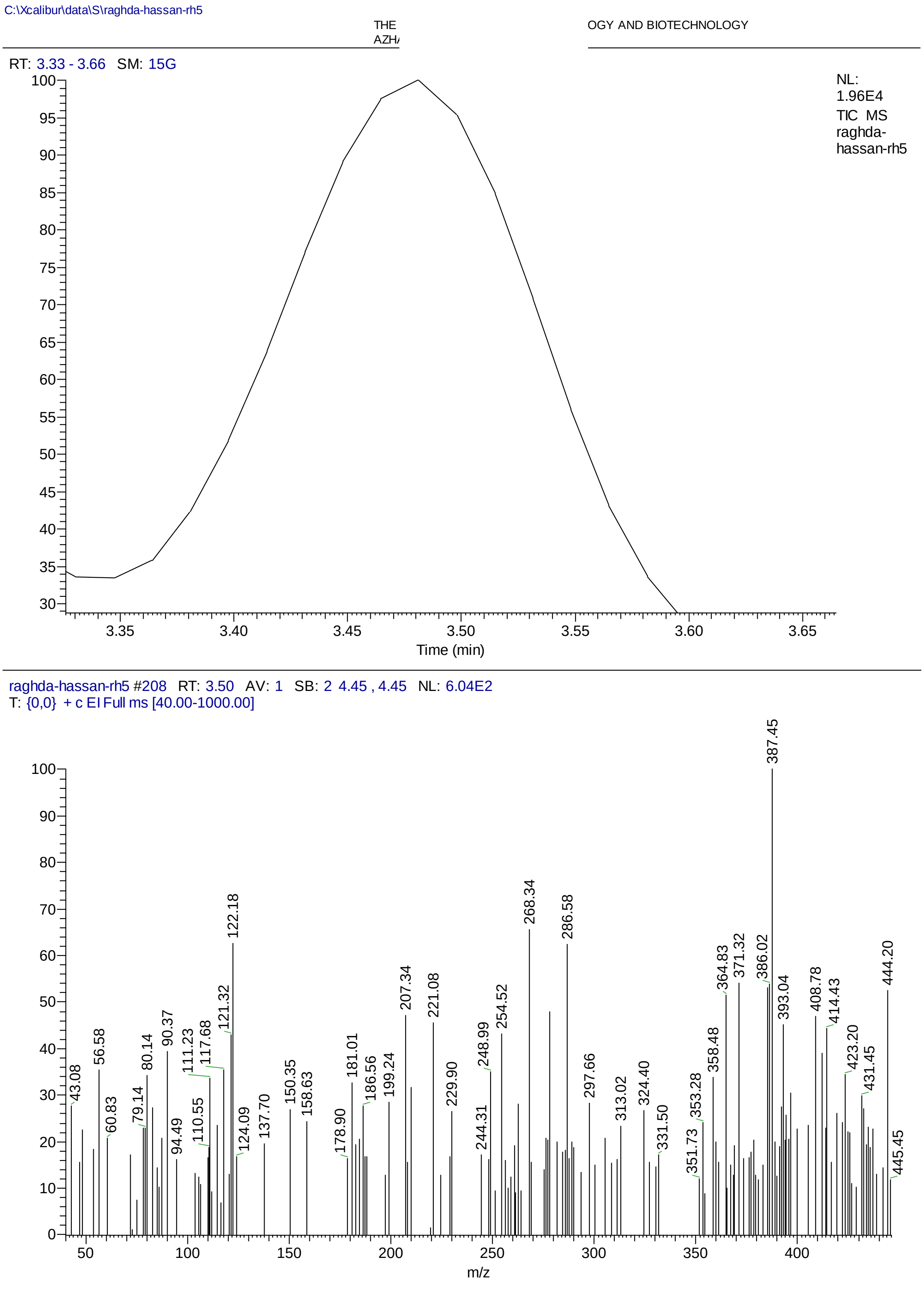

S16: Mass spectrum of compound 9f

Supporting information

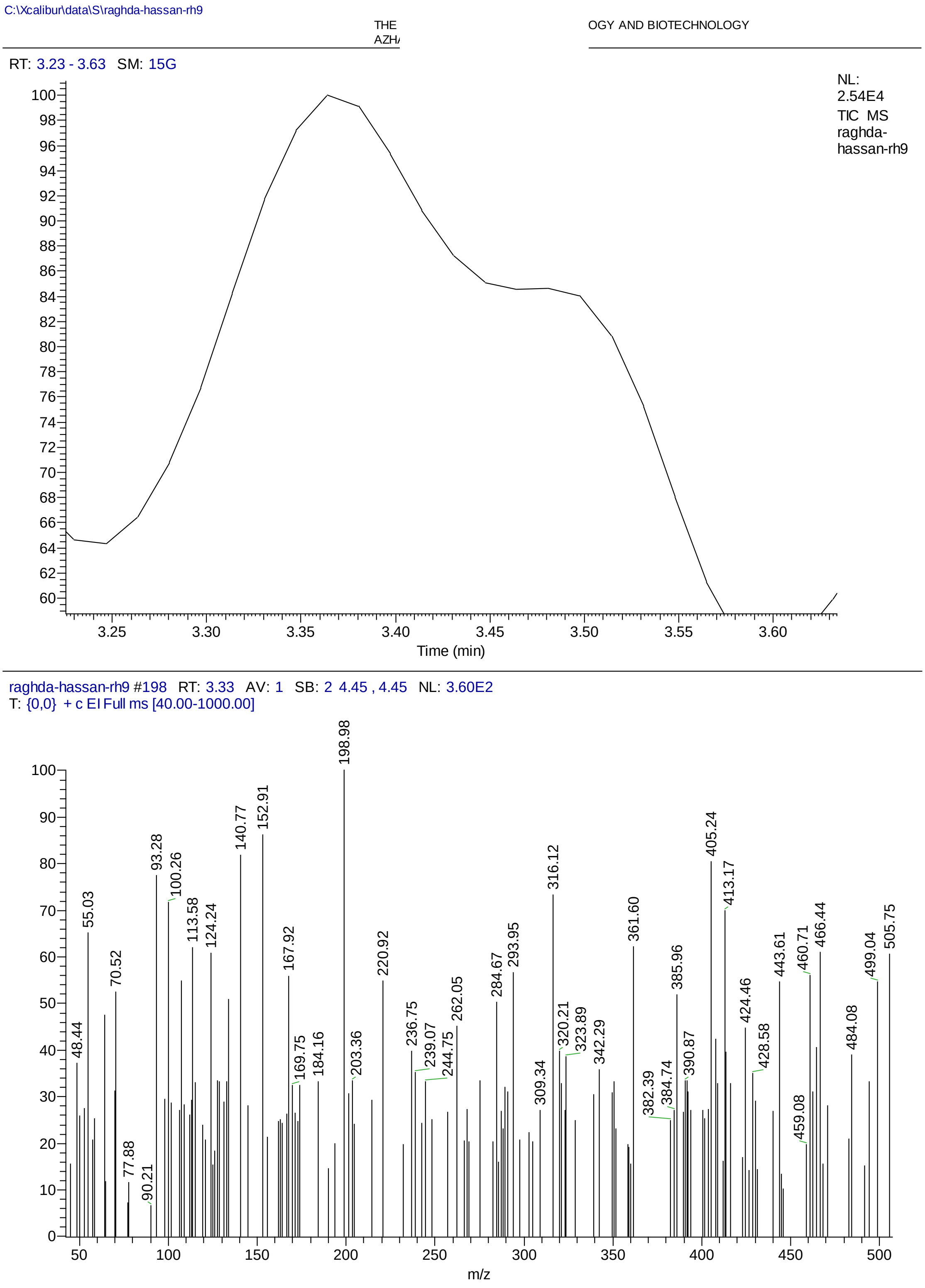

S17: Mass spectrum of compound 9g

Supporting information

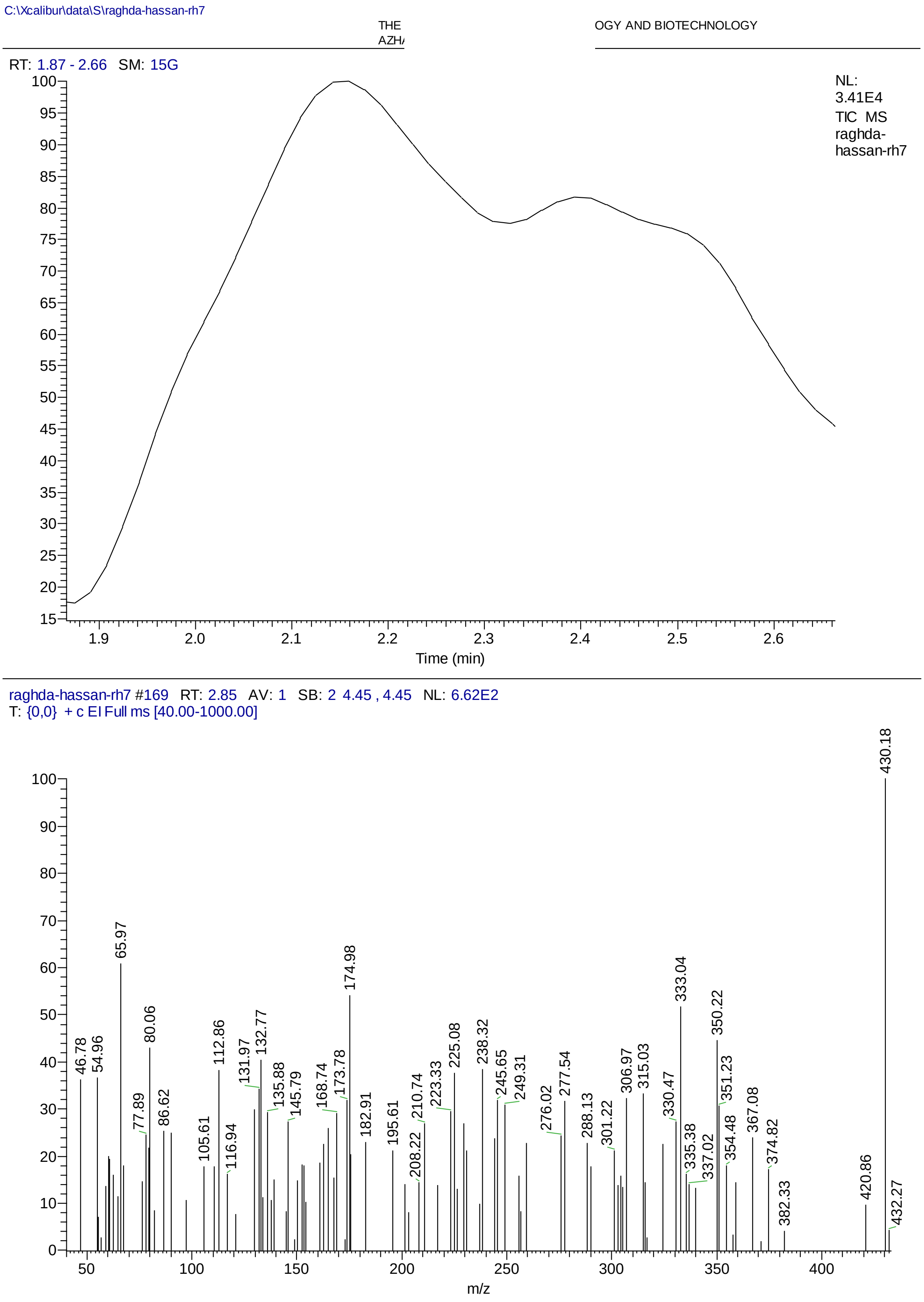

S18: Mass spectrum of compound 9h

Supporting information

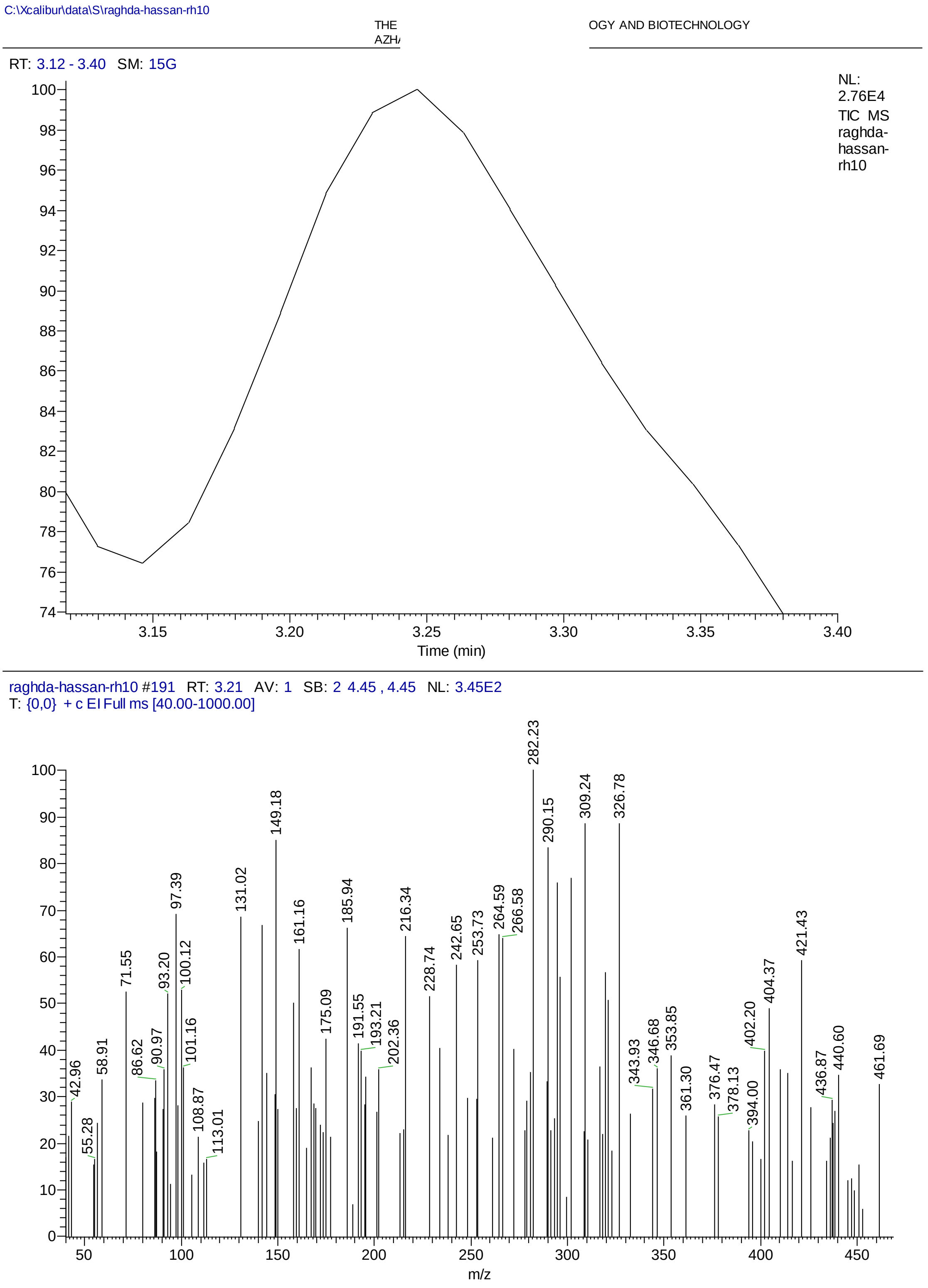

S19: Mass spectrum of compound 9i
